# Transcriptomic and Physiological Responses of *Chlorella pyrenoidosa* during Exposure to 17α-Ethinylestradiol

**DOI:** 10.3390/ijms23073583

**Published:** 2022-03-25

**Authors:** Yurui Zhang, Zixu Chen, Yue Tao, Wanyin Wu, Yuyang Zeng, Kejun Liao, Xinyue Li, Lanzhou Chen

**Affiliations:** Hubei Research Center of Environment Remediation Technology, Hubei Key Laboratory of Biomass Resource Chemistry and Environmental Biotechnology, School of Resource & Environmental Sciences, Wuhan University, Wuhan 430079, China; whdxzyr@163.com (Y.Z.); chenzixu@whu.edu.cn (Z.C.); taoyue@whu.edu.cn (Y.T.); 2019202050072@whu.edu.cn (W.W.); zyy546134918@163.com (Y.Z.); 2017301120044@whu.edu.cn (K.L.); zbh917397@163.com (X.L.)

**Keywords:** *C. pyrenoidosa*, 17α-EE_2_, photosynthetic systems, oxidative stress, transcriptomics

## Abstract

17α-ethinylestradiol (17α-EE_2_) is frequently detected in water bodies due to its use being widespread in the treatment of prostate and breast cancer and in the control of alopecia, posing a threat to humans and aquatic organisms. However, studies on its toxicity to *Chlorella pyrenoidosa* have been limited to date. This study investigated the effects of 17α-EE_2_ on the growth, photosynthetic activity, and antioxidant system of *C. pyrenoidosa* and revealed related molecular changes using transcriptomic analysis. The cell density of algae was inhibited in the presence of 17α-EE_2_, and cell morphology was also altered. Photosynthetics were damaged, while reactive oxygen species (ROS), superoxide dismutase (SOD), and malondialdehyde (MDA) content increased. Further transcriptomic analysis revealed that the pathways of photosynthesis and DNA replication were affected at three concentrations of 17α-EE_2_, but several specific pathways exhibited various behaviors at different concentrations. Significant changes in differentially expressed genes and their enrichment pathways showed that the low-concentration group was predominantly impaired in photosynthesis, while the higher-concentration groups were biased towards oxidative and DNA damage. This study provides a better understanding of the cellular and molecular variations of microalgae under 17α-EE_2_ exposure, contributing to the environmental risk assessment of such hazardous pollutants on aquatic organisms.

## 1. Introduction

Endocrine-disrupting compounds (EDCs) have been drawn much attention in the past few decades due to their endocrine-disrupting effects on humans and wildlife even at low levels [1,2]. Estrogens are a major group of EDCs that are widespread in nature and potentially harmful, causing the feminization of aquatic organisms and affecting the development of their fertilized eggs [3,4,5]. The most common synthetic estrogen is 17α-ethinylestradiol (17α-EE_2_) and it has been used as a treatment for prostate and breast cancer, chronic hemospermia, and the induction of labor since 1938 [6]. Consumption of 17α-EE_2_ is huge worldwide and it has been detected from time to time in aquatic environments in some countries in Southeast Asia, as well as in China, Brazil, Argentina, Kuwait, and Portugal [6]. Compared with natural estrogens such as estradiol (E_2_), estrone (E_1_), and 17β-estradiol (β-E_2_), 17α-EE_2_ was shown to possess more persistence and a high estrogenic activity [7]. The half-life of 17α-EE_2_ was reported to be as long as 81 days [8]. Moreover, the harm caused by 17α-EE_2_ is transmitted through the food chain and ultimately damages human health [9,10]. As a result, it was included in the EU Resolution 2015/495/EU watchlist as a priority EDC contaminant for regulation [11,12] and there is a strong need to study the biological risks and ecotoxicological effects of 17α-EE_2_.

Microalgae belong to the trophic level at the bottom end of the aquatic ecosystem and are more sensitive to pollutants, acting as important indicator species for evaluating toxicity of pharmaceuticals, nanoparticles, and organic cosmetics [13,14,15,16]. Moreover, microalgae transfer substances along the food chain and establish links with higher organisms, such as herbivorous zooplankton and herbivorous fish [17]. Previous studies showed that the exposure of estrogens could alter the algal photosynthetic system, carbohydrates, protein, and antioxidant systems [18]. Photosynthetic pigments content is reduced and the conversion of light energy is disturbed in the presence of environmental estrogens and results in the decrease in the photosynthetic efficiency. Multiple parameters, including relative reactive oxygen species (ROS), superoxide dismutase (SOD), and catalase (CAT), related to oxidative damage and antioxidants were observed to rise under the exposure of estrogens [19]. These cellular responses were usually not completely independent. For example, the growth of *Chlorella* algae was inhibited by 26% in the presence of 2 mg/L of 17β-E_2_, which also triggered oxidative stress and the decline of PSII activity [13]. A comparative study of the toxicity of various animal hormones (E_1_, 17ß-E_2_, and 17a-EE_2_, etc.) on *Chlorella vulgaris* and *Scenedesmus armatus* found that 17α-EE_2_ had the greatest effect on algal biomass and the strongest toxic effect [20]. Other studies also reported that a low concentration of 17α-EE_2_ (0.1–2 mg/L) could strongly reduce the growth of *Desmodesmus communis* and *Chlamydomonas reinhardtii* [1,21], indicating that 17α-EE_2_ poses a great threat to algal cells. Nevertheless, to our best knowledge, the systematic mechanism of the toxic effects of 17α-EE_2_ on the photosynthesis, antioxidant systems, and DNA damage repair of microalgae remains unclear.

The transcriptome analysis of algae in response to various pollutant stresses is widely used to reveal molecular changes and biological risks with good accuracy and sensitivity [22,23]. For example, exploring the toxic effects of nanoparticles, heavy metals, and microplastics on microalgae can be done by transcriptome sequencing and analyzing differential genes and metabolic pathways to reveal the mechanisms of toxicity [24,25,26]. For environmental estrogens, some advances have been made to elucidate the molecular mechanism underlying physiological indicators in exposed algae. It was found that photosynthesis- and protein-related genes participating in chlorophyll-binding, photosynthetic-antenna proteins, and photosynthetic electron transport pathways played an important role in the response to the estrogens [27]. Nonylphenols exposure could induce oxidative stress and the up-regulation of antioxidant genes in algal cells [28]. However, more detailed molecular information is needed since these known mechanisms were species-specific and concentration-specific [19,29] and should be linked to physiological changes to fully understand their significance. In addition, there is a paucity of transcriptome studies in algae exposed to 17α-EE_2_.

Previous studies found that 17α-EE2 was the most toxic hormone to *Chlorella* and significantly inhibited algal growth [20]. *Chlorella pyrenoidosa* is one of the most common single-celled microalgae with a high photosynthetic efficiency [30,31]. It reproduces rapidly, is sensitive to pollutants, and has been used as a biological model to assess the toxic effects of environmental hormones (bisphenol A and nonylphenol, etc.) [22,28]. In this study, the effects of different concentrations of 17α-EE_2_ on the physiological indicators (cell density, structure damage, photosynthetic system damage, and oxidative stress) and molecular changes (transcriptome analysis) of *C. pyrenoidosa* were synthetically investigated. It was found that low exposure concentrations mainly affected photosynthesis, while high concentrations focused on DNA damage, and oxidative stress damage occurred in all algal cells. The results of this study provide a reference for assessing the effect of 17α-EE_2_ on microalgae, thus being a valuable contribution for the environmental risk assessment of the relevant EDCs to aquatic organisms.

## 2. Results

### 2.1. The Growth of C. pyrenoidosa after Exposure to 17α-EE_2_

**Figure 1:**
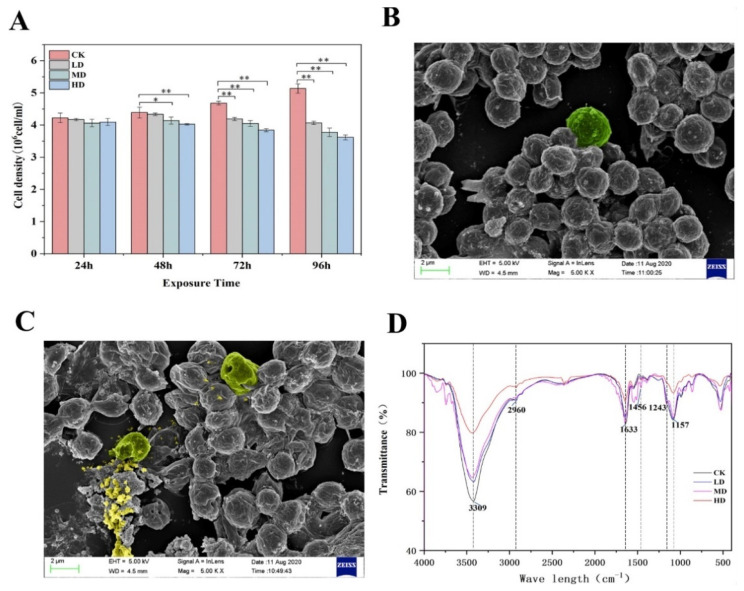
Effects of 17α-EE_2_ concentrations on the growth of *C. pyrenoidosa* within 96 h (**A**). SEM images of *C. pyrenoidosa* without 17α-EE_2_ and with 17α-EE_2_ at 96 h of exposure (**B**,**C**). FTIR analysis of *C. pyrenoidosa* cells treated with 17α-EE_2_ (**D**). * and ** represent statistically significant differences with respect to the values of control cultures at *p* < 0.05 and at *p* < 0.01 levels, respectively. CK: control; LD: low dose; MD: moderate dose; HD: high dose.

The effect of 17α-EE_2_ on the growth of *C. pyrenoidosa* is shown in Figure 1A. There was no significant hormonal effect on the growth of *C. pyrenoidosa* in the 17α-EE_2_ group compared to the control group during the 24 h of 17α-EE_2_ treatment. At 48 h, there was no significant decrease in cell density in the LD group, while the growth of *C. pyrenoidosa* decreased in a dose-dependent manner in the MD and HD groups. Growth was significantly inhibited in the MD and HD groups, with a cell density of only 94.32% and 91.72% of that in the control group, respectively. At 72 h and 96 h, the cell density decreased in all the treatment groups, with that in the LD, MD, and HD groups being 89.47%, 86.53%, and 82.02% of that in the control group at 72 h, respectively. At 96 h, the cell density in these groups was only 79.17%, 73.51%, and 70.41% of that in the control group.

### 2.2. Characterization by SEM and FTIR Spectroscopy

As shown in the SEM results, the control group exhibited an intact cell morphology with no damage to the cell wall in the 96 h period (Figure 1B). In contrast, within 96 h of 17α-EE_2_ stress, the algal cells appeared to be agglomerated, and the integrity of the cell membrane was disrupted, resulting in the outflow of the cell contents (Figure 1C).

FTIR spectroscopy analysis was used to determine the dynamic relative changes in the levels of proteins, carbohydrates, lipids, and other biomolecules in *C. pyrenoidosa* cells exposed to 17α-EE_2_ (Figure 1D). At 96 h, the transmittance of the peaks at 3309 cm^−1^, 2960 cm^−1^, and 1633 cm^−1^ was greater in the treatment group than in the CK group, with the peak at 3309 cm^−1^ representing the stretching vibration of the N–H bond in proteins and the O–H bond in carbohydrates. The peak at 2960 cm^−1^ represents the –CH_3_ antisymmetric stretching vibration in proteins, carbohydrates, and lipids, and that at 1633 cm^−1^ represents the amide *I* of the β-sheets, indicating a decrease in the levels of biomolecules, such as proteins and lipids, and the MD group decreased more compared with other treatment groups. The peak for the C=O stretching vibration in the protein carboxylic acid group appeared at 1456 cm^−1^, and the intensity of the peak was higher in all treatment groups than in the CK group, but it was highest in the MD group than in the other groups. The peak at 1243 cm^−1^ was mainly derived from the nonvibrational stretching vibrations of the P=O group of phospholipids, with a decrease in peak intensity compared to the CK group indicating a decrease in intracellular phospholipid content, with the greatest decrease in the MD group. The absorption peak associated with the sugar skeleton at 1157 cm^−1^ had the highest passage rate in the CK group, while the HD group had a higher transmission than the other treatment groups, indicating that sugar synthesis in the algal cells was affected.

### 2.3. Effects on Photosynthesis

**Figure 2:**
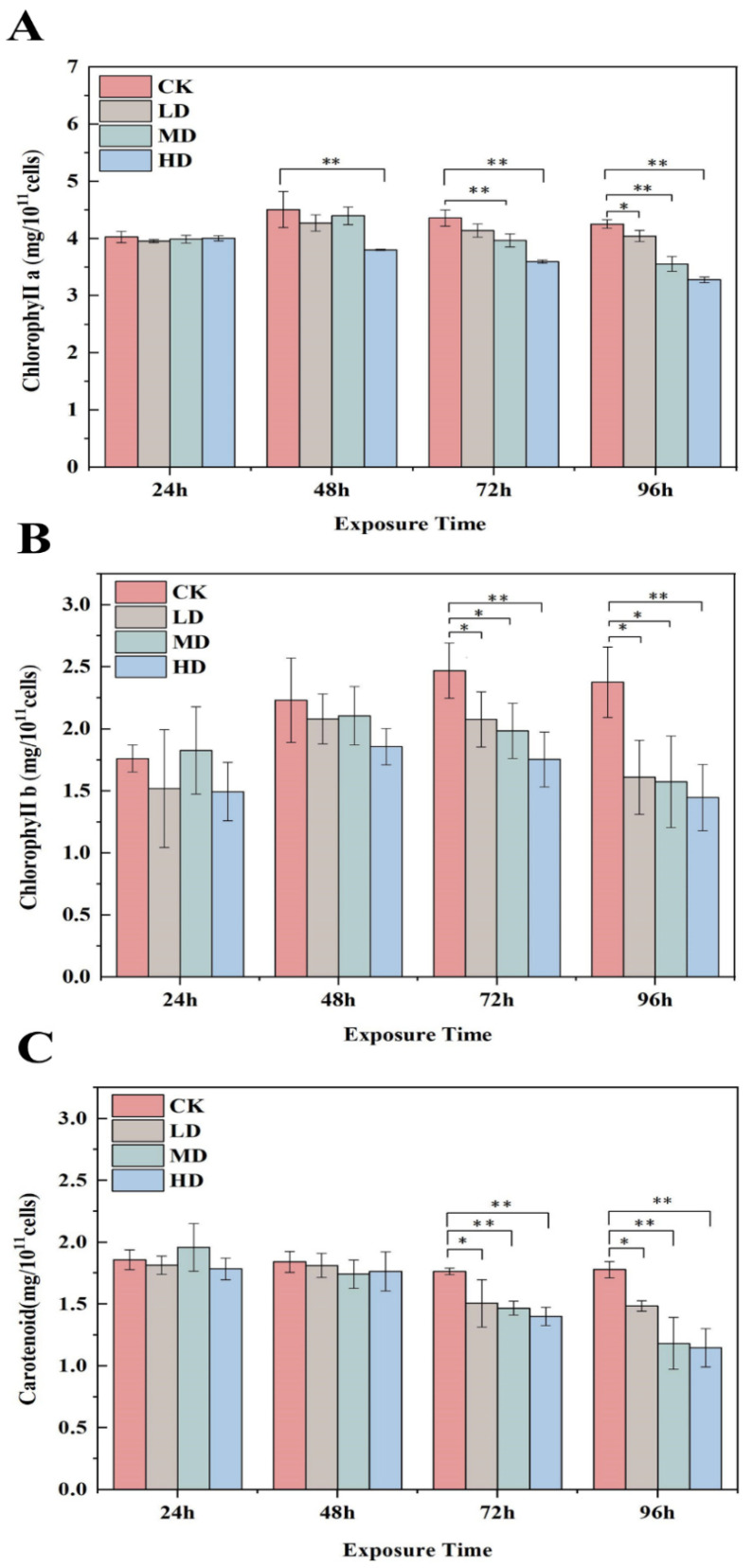
Effects of 17α-EE_2_ concentrations on *C. pyrenoidosa* chlorophyll a, b (**A**,**B**) and carotenoid (**C**) content within 96 h exposure. * and ** represent statistically significant differences with respect to the values of control cultures at *p* < 0.05 and at *p* < 0.01 levels, respectively. CK: control; LD: low dose; MD: moderate dose; HD: high dose.

The chlorophyll a and b and carotenoid levels of *C. pyrenoidosa* were affected by exposure to 17α-EE_2_ for 96 h, as shown in Figure 2A–C. There was no obvious pattern of change in the chlorophyll content relative to the CK group within 24 h, but the chlorophyll a and b levels both began to decrease between 48 and 72 h, with both being lower than the level in the CK group, and the inhibition increased with increasing 17α-EE_2_ concentrations. The higher the treatment concentration was, the lower the chlorophyll content. There were no significant differences in carotenoid content up to 48 h. From 72 h onwards, there was a significant decrease in all treatment groups. The carotenoid content decreased by 10.53%, 13.47%, and 17.98%, respectively, at 72 h in comparison with the CK group, while the decreases were 20.83%, 26.49%, and 29.59% at 96 h of treatment, respectively.

**Figure 3:**
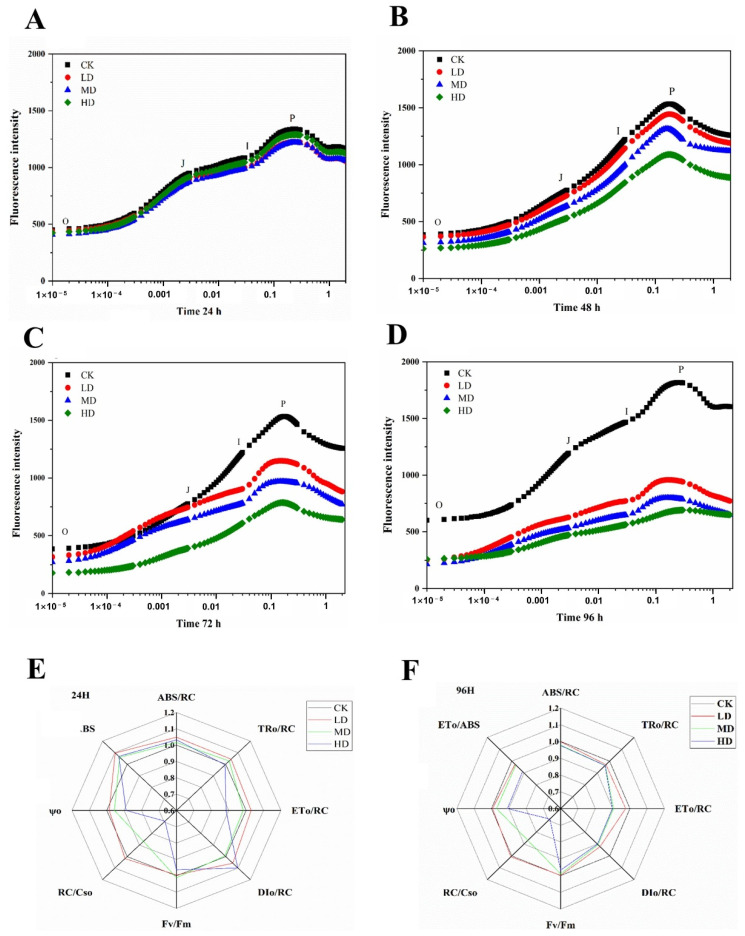
Effects of 17α-EE_2_ concentrations on the chlorophyll a fluorescence transients (**A**–**D**) of *C. pyrenoidosa* within 96 h exposure and chlorophyll fluorescence parameter radar diagram at 24 h, 96 h (**E**,**F**). CK: control; LD: low dose; MD: moderate dose; HD: higher dose.

The effects of different concentrations of 17α-EE_2_ on the photosynthetic system of *C. pyrenoidosa* during exposure were investigated in this paper, and the results are shown in Figure 3. The rapid chlorophyll fluorescence-induction kinetics indicated the change in fluorescence from the O to P points, reflecting changes in the primary photochemical reaction of photosystem II (PSII) and the structure and state of the photosynthetic machinery. The chlorophyll fluorescence parameters mainly reflect the absorption, conversion, utilization, and distribution of light energy in plants and can reflect the photochemical reaction activity in algal cells and their self-protection ability in real time (Table S1). The chlorophyll fluorescence curves (OJIP curves) of the leaves and their parameters changed significantly after 96 h of treatment with different concentrations of 17α-EE_2_ (Table S2), indicating that 17α-EE_2_ severely disrupted the photosynthetic organ structure and function of the leaves. K-steps occurred at both 72 h and 96 h, with even more inflection points occurring in this period. The Fv/Fm of *C. pyrenoidosa* was inhibited irrespective of 17α-EE_2_ concentration and exposure time. The photosynthetic activity of the algae decreased with increasing concentrations of 17α-EE_2_, and the higher the concentration was, the more rapidly the activity decreased. Different concentrations of 17α-EE_2_-stress significantly reduced the corresponding photosynthetic parameters (ABS/RC, RC/CS_0_, ET_0_/ABS, ψ_O_, RC/CS_0_, DI_0_/RC, ET_0_/RC, and TR_0_/RC) and inhibited the photosynthetic activity of *C. pyrenoidosa* over a 96 h period.

### 2.4. Variation in the MDA Levels, SOD Activity, and ROS Generation in Response to 17α-EE_2_

**Figure 4:**
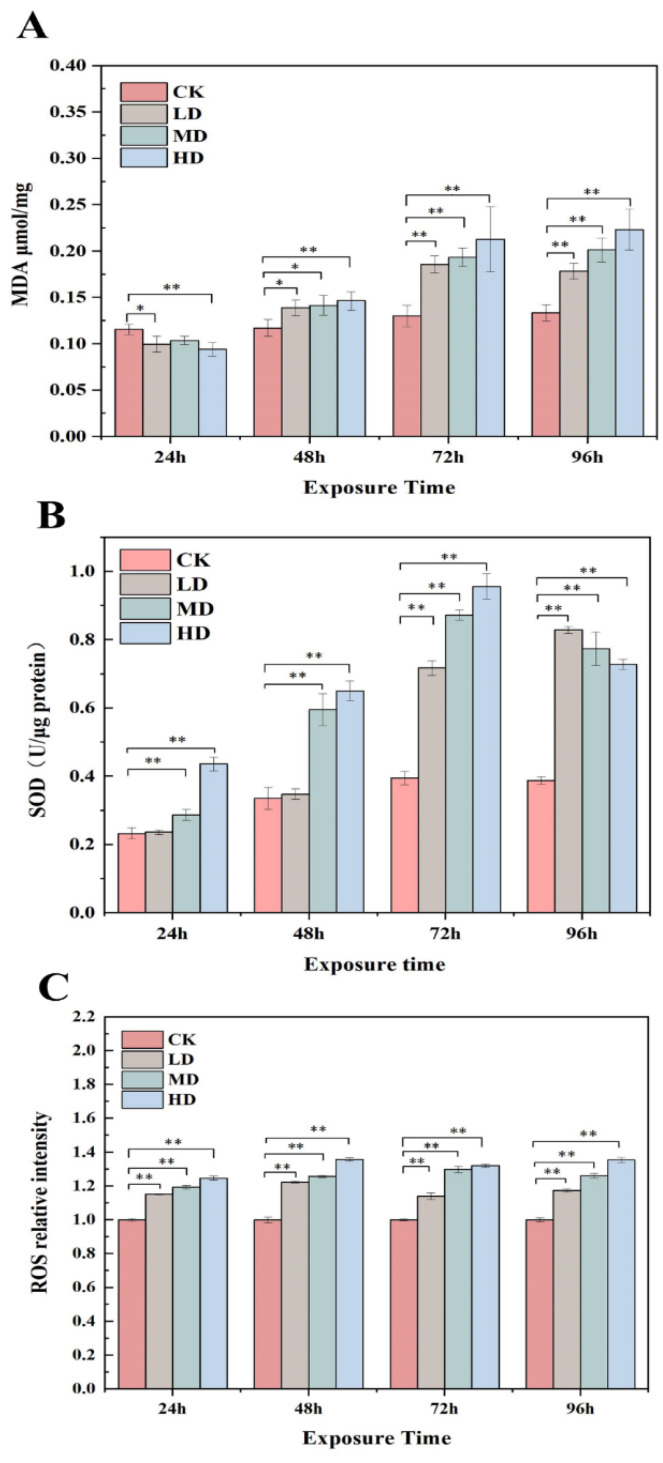
Effects of 17α-EE2 concentrations on MDA content (**A**), SOD activity (**B**), and ROS activity (**C**) in *C. pyrenoidosa* within 96 h exposure. * and ** represent statistically significant differences with respect to the values of control cultures at *p* < 0.05 and at *p* < 0.01 levels, respectively. CK: control; LD: low dose; MD: moderate dose; HD: higher dose.

MDA, as one of the end products of cellular lipid peroxidation, is often used to characterize whether lipid peroxidation occurs in cell membranes under environmental stress. Figure 4A shows the changes in the MDA content of *C. pyrenoidosa* cells in response to different 17α-EE_2_ exposure concentrations and durations. The results showed that the MDA content of the CK group remained stable and relatively low over time. The MDA content of algal cells in the treated groups increased with the addition of 17α-EE_2_ from 48 h onwards. At 72 h, the MDA content of algal cells in the LD, MD, and HD treatment groups increased by 37.57%, 43.30%, and 57.55%, respectively, compared to that of the CK group under the effect of 17α-EE_2_. After 72 h of exposure, the percentage increase in MDA content decreased over time in the LD group, while it continued to increase in the MD and HD groups. The MDA content increased by 47.26% and 63.20% after 96 h of exposure in the MD and HD groups, respectively, compared to the CK group; however, it only increased by 30.43% in the LD group.

The intracellular antioxidant enzyme system was able to regulate intracellular reactive oxygen radicals and reduce peroxidative damage to cells. In this study, changes in SOD activity in *C. pyrenoidosa* were examined in the presence of 17α-EE_2_ (Figure 4B). The SOD activity in algal cells in the CK group remained basically stable, and the SOD content in algal cells at different time points was higher at different concentrations of 17α-EE_2_ than that in the CK group. At 24 h, the SOD content increased significantly in the MD and HD groups. The SOD activity continued to increase in all three groups from 48 to 96 h, with the highest value occurring at 72 h when the activity in the HD group increased by 0.56 U/μg protein compared to that in the CK group.

DCFH is oxidized at high fluorescence intensities and is itself nonfluorescent. Intracellular ROS and other peroxides cause DCF production, so ROS production can be measured by determining the fluorescent DCF level. The increase in ROS and MDA levels was not consistent. There was a greater degree of ROS production at all concentrations, with the stress increasing over time, but the maximum ROS stress level remained at approximately 1.36 times that of the control (Figure 4C).

### 2.5. Differentially Expressed Genes

**Figure 5:**
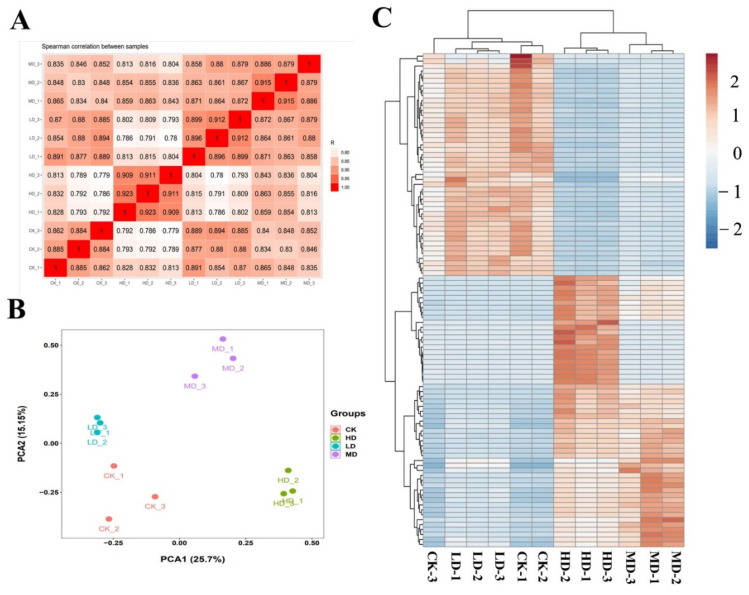
Transcriptomic profiling of *C. pyrenoidosa* after 96 h exposure to 17α-EE_2_. Correlation analysis of patterns of gene expression in control and 17α-EE_2_-treated groups (**A**); principal component analysis (PCA) of FPKM profiles in 17α-EE_2_-treated groups (**B**); heat map of differential gene-clustering analysis (**C**). CK: control; LD: low dose; MD: moderate dose; HD: higher dose.

**Figure 6:**
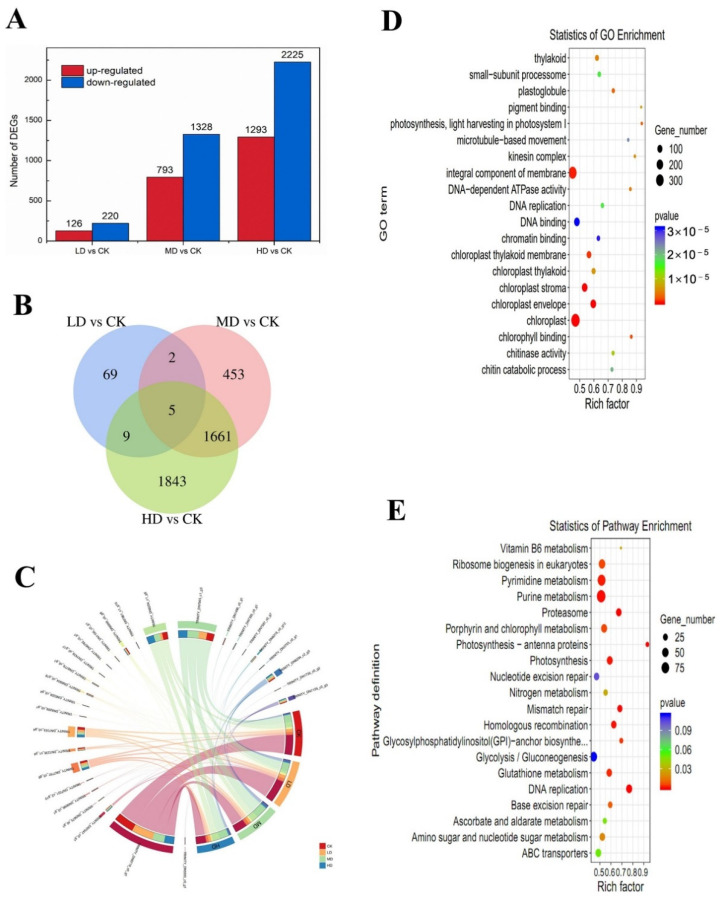
Differential gene expression after 17α-EE_2_ exposure (**A**); Venn diagram of the number of DEGs in each 17α-EE_2_-treated group (**B**); chord diagram representing gene abundance (**C**); scatter plots of GO function enrichment in each group (**D**); scatter plots of KEGG enrichment pathway in each group (**E**). CK: control; LD: low dose; MD: moderate dose; HD: higher dose.

A total of 24,241 transcripts were detected in *C. pyrenoidosa*. The correlation among the three replicate-gene-expression patterns of each group was significant (Figure 5A). Principal component analysis (PCA) showed that the gene-expression clusters in the control, LD, MD, and HD groups were different (Figure 5B), which was consistent with the results of the heatmap assay (Figure 5C). We identified 346 (126 up- and 220 down-regulated), 2121 (793 up- and 1328 down-regulated), and 3518 (1293 up- and 2225 down-regulated) differentially expressed genes (DEGs) in each group after low-, medium-, and high-level exposure to 17α-EE_2_, respectively (Figure 6A). The Venn diagram shows the number of identical and unique DEGs between different concentration groups (Figure 6B), and circle plots show the gene abundance (Figure 6C). The number of DEGs increased significantly with increasing stress concentration, with the HD group having the highest number of DEGs. To validate the RNA-Seq results, the expression levels of three DEGs from different pathways were measured by qRT-PCR (Table S3). The results showed that the direction and size of the changes in the mRNA expression of these genes were consistent with the results of the transcriptome analysis (Figure S1).

### 2.6. Analysis of Transcriptional Changes

**Figure 7:**
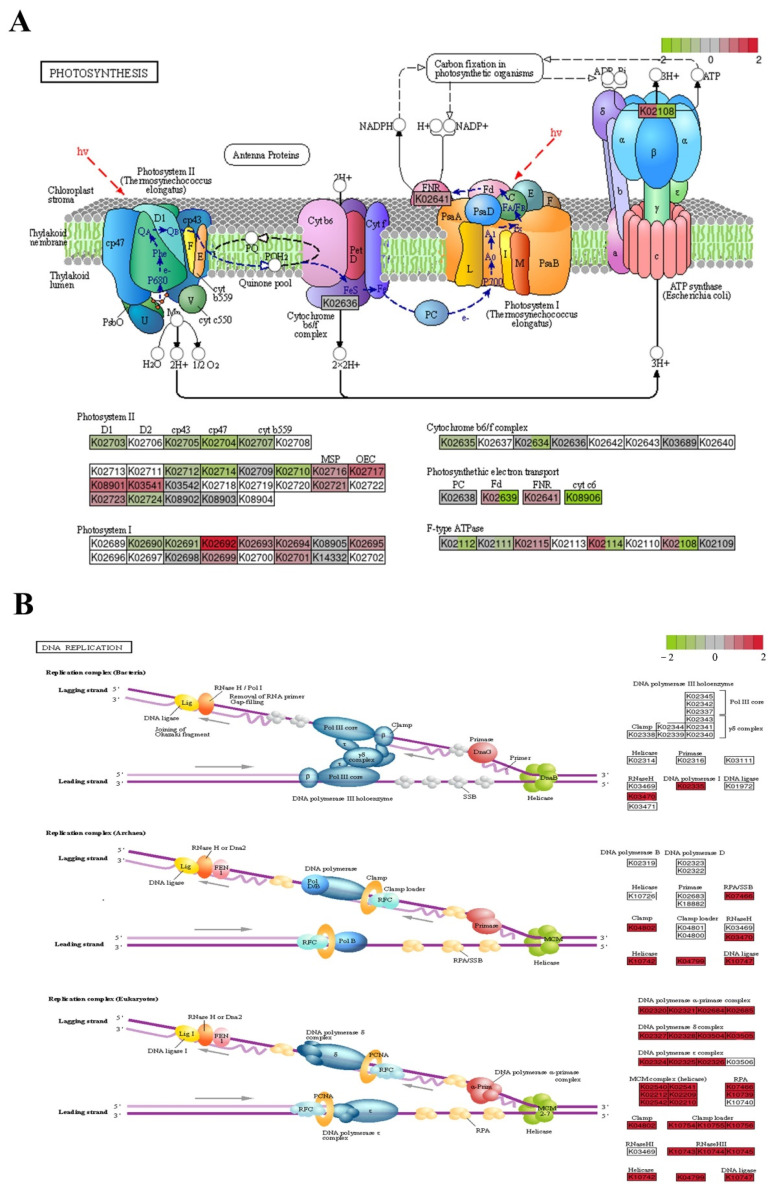
Changes in expression of KEGG-annotated genes in photosynthesis and DNA replication pathways (**A**,**B**); red squares: significantly up-regulated in the treatment group compared to CK, green squares: significantly down-regulated in the treatment group compared to CK. CK: control; LD: low dose; MD: moderate dose; HD: higher dose.

After extensive analysis of the transcriptome, changes in the expression of specific pathways and transcription factors were investigated. Upon combining the top 20 enriched pathways from the GO and KEGG analysis results (Figure 6D,E), DNA synthesis and repair and photosynthesis were found to be the most significantly enriched pathways (with 109 and 88 down-regulated and seven and 11 up-regulated, respectively). The KEGG annotation of the photosynthesis-related metabolic pathways, and changes in gene expression in response to DNA damage are shown in Figure 7A,B. Genes related to photosynthesis (*petJ*, *psaD*, *psbB*, and *psbI*) and the carotenoid-regulated gene *crtz* were down-regulated (Table S4). While in the DNA replication pathway, genes such as *mcm4* significantly down-regulated and *rpa1D* genes responsible for mismatch repair were significantly up-regulated. Several specific pathways exhibited various behaviors at different levels (Figure S2). Ribosome biogenesis in eukaryotes, photosynthesis, and oxidative phosphorylation were the three most significantly enriched pathways (22 genes significantly down-regulated and six genes significantly up-regulated, *p* < 0.05) in the KEGG analysis of the LD group compared to the CK group (Figures S3–S5). The genes in the photosystem II pathways, *psbA*, *psbB*, *psbC*, and *psbE* were significantly down-regulated while *psb28* was up-regulated. The down-regulated genes *atpA*, *atpG*, and *cox1* were related to oxidative phosphorylation. When the MD group was compared with the CK group, pyrimidine metabolism, ribosome biogenesis in eukaryotes, porphyrin and chlorophyll metabolism, nitrogen metabolism, and DNA replication were the pathways that were found to have the most enriched and significant genes, with a total of 102 significant DEGs (*p* < 0.05) (Figures S6–S10). Changes in the expression of KEGG-annotated genes in nitrogen metabolism pathways showed that the down-regulated genes *gdh2* and *kin14n* were related to the nitrogen cycle and ribosomal protein. In response to oxidative damage, the genes *sodA* and *por* were up-regulated. When the HD group was compared to the CK group, the most important pathways were DNA replication and mismatch repair and homologous recombination; 87 of the 89 DEGs were down-regulated, algal cell growth was severely inhibited, and the DNA damage was severe (Figures S11–S13). The genes on the mismatch repair pathway, such as *msh1*, were up-regulated, while the others, such as *exo1* and *mus1*, were significantly down-regulated.

## 3. Discussion

Exposure to 17α-EE_2_ inhibits the growth of *C. pyrenoidosa* and disrupts the cell structure. The reduction in chlorophyll content in algal cells leads to less efficient photosynthesis in microalgae, which can lead to a lack of optical energy and thus the suppression of the cellular energy transfer and metabolism, which can ultimately inhibit algal growth [32]. Changes in the carotenoid content in algal cells exposed to 17α-EE_2_ are among the mechanisms by which algae resist environmental stress; carotenoids are photoprotective during photosynthesis, and their biosynthesis is a direct indicator of photosynthetic capacity [24]. Carotenoids can act as antioxidants to mitigate oxidative damage [33]. These protective mechanisms allow *C. pyrenoidosa* to remain active after 17α-EE_2_ stress; however, the rise in carotenoid levels only briefly counteracts the hormonal effects, after which they remain inhibited, leading to a reduction in photosynthetic activity. Carotenoids exert antioxidant activity by quenching the excessive intracellular accumulation of ROS, protecting against photodamage caused by quenching singlet oxygen and trilinear chlorophyll, and dissipating excess absorbed energy by reacting with excited chlorophyll molecules to reduce the extent of cellular lipid peroxidation [34]. The variation in the chlorophyll content was greater than that in the carotenoid content because of the higher sensitivity to oxidative damage and greater susceptibility to breakdown by ROS under environmental-stress conditions [35].

The chlorophyll fluorescence curves (OJIP curves) of *C. pyrenoidosa* and their parameters changed significantly after 1 h of treatment with different 17α-EE_2_ levels, indicating that 17α-EE_2_ severely disrupted the photosynthetic organ structure and function of the algal cells. Duress leads to the appearance of the K-step (300 μs), which is caused by the inhibition of the hydrolysis system and the inhibition of part of the receptor side prior to QA, during which it is the oxygen emission complex (OEC) that is injured, so the K-point can be used as a specific marker of OEC injury. Reduced photosynthetic quantum production due to the attenuation of the OEC state impairs photosynthesis in algal cells [36]. PSII is the first pigment protein complex in the electron transport chain in the chloroplast vesicle-like membrane, consisting of more than 20 protein subunits. It catalyzes the light-driven cleavage of water and the oxidation of quinones [37]. In this study, the PSII activity (Fv/Fm) of the algal cultures decreased significantly with increasing concentrations of 17α-EE_2_, and decreases in parameters such as RC/CSo and ψ_O_ suggested that inhibition led to the closure of PSII reaction centers [38]. At the same time, the fluorescence yield and photosynthetic electron transport were reduced, mainly due to oxidative damage caused by increased ROS levels in the body.

The production and accumulation of substances within microalgae cells are susceptible to environmental influences. Under stress conditions, cells are able to respond to adverse growth factors by altering the content of cellular macromolecules [39]. Exposure to 17α-EE_2_-induced oxidative stress was confirmed by increased levels of ROS and MDA in cells and altered antioxidant enzyme activity. The elevation in the MDA content occurred due to the imbalance of the free radical metabolism and the accumulation of ROS in algal cells, which may have caused or exacerbated membrane lipid peroxidation, resulting in a significant increase in MDA content [32]. Excessive production of ROS in algal cells may occur due to the inhibition of photosynthesis, enhancing excess excitation energy in chloroplasts [27]. The excessive accumulation of ROS is an important source of photosynthetic pigment damage, and if the damage is severe, the photobleaching of plants can occur (Miller et al., 2010). SODs are the first antioxidant defense system of the cell when plants are exposed to oxygen stress. The three most common types of SODs in plants are Mn-SOD, Cu/Zn-SOD, and Fe-SOD, the main function of which is to scavenge O^2−^ [40]. The increase in SOD activity may have occurred due to the progressive production of superoxide, which is thought to be a central component of signal transduction, the level of which increases with the activation of the enzyme pool or with increased SOD gene expression [41]. Some oxidative damage was caused to *C. pyrenoidosa*, and the inhibition and damage increased with increasing hormone concentrations and treatment times.

Treatment with 17α-EE_2_ altered gene expression in *C. pyrenoidosa*, which can respond to 17α-EE_2_ toxicity by up-regulating or down-regulating some genes to maintain normal growth. The 17α-EE_2_ was found to have a significant inhibitory effect on genes regulating algal growth, photosynthetic pigments, photosynthetic efficiency, antioxidant systems, and DNA replication after 96 h of exposure. At the transcriptional level, algal cells mitigate damage and maintain growth by activating repair mechanisms, a process that includes the repair and replacement of reaction center proteins and the up-regulation of ROS scavenger gene expression, corroborating biochemical phenomena. In the previous study, *Microcystis aeruginosa* (*M. aeruginosa*) under a mixed antibiotic treatment up-regulated three genes targeting ROS scavenging (*MAE_36510*, *MAE_58300*, and *trxA*). During redox, reactive oxygen species scavenging genes were enriched to maintain cyanobacterial cell redox homeostasis. In addition, the up-regulation of photosynthesis-related genes may help to increase the photosynthetic activity of antibiotic-treated algal cells, thereby promoting the growth of *M. aeruginosa* through increased photosynthetic energy production [42].

In algal cells, gene regulation changes with increasing stress levels, with higher stress leading to more severe damage. Low concentrations of 17α-EE_2_ mainly disrupt the photosystem, impede electron transfer, and affect energy conversion. During the photoelectron transfer process, both *petB* and *petF* were also significantly down-regulated (Table S4), while in previous research, the expression of the genes *petF* and *petH*, which are related to the regulation of photosynthetic electrons, and the two PSII subunit genes *psbO* and *psbP*, was increased in the irradiated mutants of *Nannochloropsis oceanica*, leading to enhanced electron transfer during the photoreaction and possibly accelerating the water cleavage process and the rate of oxygen production in the mutants [43].

Genes related to photosynthesis (*petJ*, *psbB*, and *psbI*) were down-regulated in the medium- and high-level treatment groups, and the carotenoid-regulated gene *crtz* was down-regulated. Nitrogen metabolism pathways in the medium-concentration groups, *nita* and *gdh2*, were down-regulated, and the *kin14n* genes in the ribosome biosynthesis were significantly down-regulated. Algal cell growth will be affected by the reduction of carbon and nitrogen compounds. Similar phenomena have been found in previous studies. *Chromochloris zofingiensis* cultured in heterotrophic conditions in the dark demonstrated that the “lipid biosynthesis proteins” and “ribosome biosynthesis” pathways are significantly down-regulated and that the biosynthesis of major C-containing compounds (lipids, starch, and proteins) is inhibited when algal cells are exposed to energy limitation [44].

The most severe effect was DNA damage, with genes such as *mcm4* being significantly down-regulated and with *rpa1D* genes responsible for mismatch repair significantly up-regulated. DNA damage is one of the most severe forms of oxidative stress, and its repair is linked to cell survival and prognosis [45]. DNA mismatch repair prevents the occurrence of permanent mutations during cell division by correcting the DNA mismatches that occur during DNA replication. Similar DNA damage was observed when transcriptome analysis was performed on erythromycin-stressed *Raphidocelis subcapitata* [46]. *Dunaliella salina* shows the up-regulation of most DNA replication-related genes in the presence of molybdenum disulfide nanoparticles (key genes such as DNA polymerase alpha subunit A, *mcm6*), in order to promote cell division and overcome environmental stresses [47]. It is concluded that 17α-EE_2_ primarily impaired the photosynthesis of *C. pyrenoidosa*, then induced antioxidant system response and DNA damage as its concentration increases.

## 4. Materials and Methods

### 4.1. Algal Cultures

The freshwater green microalga *C. pyrenoidosa* (No. FACHB-5) was purchased from the Institute of Hydrobiology, Chinese Academy of Sciences (Wuhan, China). The algal cells were transferred aseptically to freshly sterilized BG11 medium (pH = 7.1). Then, they were incubated in a light incubator at a constant temperature of 25 ± 1 °C with a light intensity of 40 μE m^−2^ s^−1^ and a 12 h:12 h alternating light/dark light cycle until the logarithmic phase of growth. The algal cultures were shaken at least three times a day to keep the algal cells in suspension and to promote the transfer of carbon dioxide [22].

### 4.2. 17α-EE_2_ Exposure

The 17α-EE_2_ was dissolved in dimethyl sulfoxide and sonicated for 1 h. The stock solution was obtained and then sterilized by membrane filtration (average pore size, 0.2 μm). Then, it was diluted with sterilized culture medium to achieve a series of concentrations, including 0 mg/L (control), 2 mg/L (low dose), 4 mg/L (moderate dose), and 8 mg/L (high dose). The initial absorbance at 680 nm of the *C. pyrenoidosa* culture was approximately 0.13, and the cell density was approximately 1.80 × 10^6^ cells/mL. Three independent assays were accomplished for each treatment group. The culture was incubated for 96 h with the above culture conditions and shaken three times a day to ensure that the cells were well mixed.

### 4.3. Determination of Cell Density and Photosynthetic Pigment Content

The growth of *C. pyrenoidosa* was measured every 24 h using a UV/VIS spectrophotometer (UV2204PC, Shanghai Jipu Instrument Co. LTD, Shanghai, China) to determine the absorbance at 680 nm. The cell density was estimated by hemocytometry under a microscope. A standard curve and regression equation were established between absorbance values and algal density: cell density (10^6^ cells/mL) = 3.6831 × OD680 − 0.652 (R^2^ = 99%).

Photosynthetic pigment measurements were carried out using methods that have been described elsewhere [34]. Algal cultures were centrifuged at 8000 rpm for 10 min. Then, the supernatant was discarded, and the algal bodies were collected. Five milliliters of 95% ethanol were added, and the algal cells were extracted for 24 h in a refrigerator at 4 °C and shaken 2–3 times to allow the cytochromes to fully dissolve. The supernatant was centrifuged (8000 rpm, 10 min), and the absorbance values D665, D649, and D470 were determined spectrophotometrically to calculate the chlorophyll a and b and carotenoid levels.

### 4.4. Measurement of Chlorophyll Fluorescence

The chlorophyll fluorescence parameters of *C. pyrenoidosa* were measured every 24 h with a portable plant efficiency analyzer (PEA, Hanstech, UK). The OJIP test analysis was performed according to available methods and described as follows: samples were placed in the instrument after 15 min of dark adaptation and were measured after 5 s of recording time. The measurements were carried out at room temperature with an excitation light intensity of 50% of the maximum light intensity (1500 μE/m^2^ s) [2,48]. The fluorescence parameters were calculated by a previous study [49].

### 4.5. Analysis of Contents of MDA, SOD, and ROS

The MDA and SOD levels were measured within 96 h. A total of 5 mL of fresh algal solution was centrifuged (8000 rpm, 10 min), the algal bodies were collected and washed three times with phosphate buffer (0.1 mol/L, pH 7.8), and 5 mL of phosphate buffer (0.1 mol/L, pH 7.8) was added. The mixture was ultrasonicated in an ice bath and centrifuged at low temperature, and the supernatant was used as the crude enzyme extract for testing. To measure the MDA content, 2 mL 10% trichloroacetic acid and 2 mL 0.6% thiobarbituric acid were added to the crude enzyme solution. The mixed liquor was then incubated in boiling water bath for 20 min, and the supernatants were measured at absorbances of 440, 532, and 600 nm after centrifugation. The nitro blue tetrazolium photoreduction method was used to determine SOD, and absorbance values at 560 nm were measured and calculated [50].

ROS generation was analyzed by fluorescence spectrophotometry (dichloro–dihydro–fluorescein diacetate (DCFH-DA) method) [51]. The culture samples were centrifuged at 8000 rpm and 4 °C for 10 min. The supernatants were discarded and the cell pellets were resuspended with pure water. DCFH-DA was then added, and the solutions were shaken before being incubated in the dark for 30 min. The fluorescence content of the algal samples was measured using a fluorescence spectrophotometer (F-4500, Hitachi, Tokyo, Japan) with an excitation wavelength of 485 nm and emission wavelength of 535 nm.

### 4.6. Morphological Examination

The morphological characteristics of the algal cell surface after exposure to 17α-EE_2_ were identified using field emission SEM (Zeiss SIGMA, Cambridge, UK) and observed according to the method described in a previously reported method [52]. Algae exposed for 24 h were selected, and the algal solution was pretreated, freeze-dried, and coated with gold for SEM observation.

### 4.7. Determination of Surface Characteristics

Fourier transform infrared (FTIR) spectroscopy analysis was used to identify changes in biomolecules, including proteins, carbohydrates, and lipids, within algal cells under 17α-EE_2_ exposure to assess the extent of cellular damage. The samples and associated spectra were analyzed according to the available method [53].

### 4.8. Transcriptome Sequencing Analysis

Total RNA was extracted using TRIzol reagent (Invitrogen, Carlsbad, CA, USA) following the manufacturer’s procedure. Each sequenced sample was subjected to 3 biological replicates, and we performed paired-end sequencing on an Illumina NovaSeq™ 6000 instrument (LC Sciences, Houston, TX, USA). First, in-house Cutadapt [54] and Perl scripts were used to remove the readings that contained adaptor contamination, low-quality bases, and unidentified bases. Then, sequence quality was verified using FastQC (http://www.bioinformatics.babraham.ac.uk/projects/fastqc/ (accessed on 21 March 2022)), including the Q20, Q30, and GC content of the clean data. All downstream analyses were based on clean data of high quality. De novo assembly of the transcriptome was performed with Trinity 2.4.0 [3,55]. Annotation of assembled genes was performed by using public databases. All the assembled unigenes were aligned against the nonredundant (Nr) protein (http://www.ncbi.nlm.nih.gov/ (accessed on 21 March 2022)), Gene Ontology (GO; http://www.geneontology.org (accessed on 21 March 2022)), SwissPr 23 March 2022ot (http://www.expasy.ch/sprot/ (accessed on 21 March 2022)), Kyoto Encyclopedia of Genes and Genomes (KEGG; http://www.genome.jp/kegg/ (accessed on 21 March 2022)), and EggNOG (http://eggnogdb.embl.de/ (accessed on 21 March 2022)) databases using DIAMOND [56] with a threshold of E value < 0.00001. The differentially expressed unigenes were selected with the criterion log2 (fold change) > 1 or log2 (fold change) < −1 and with statistical significance (*p* < 0.05) by R package edgeR (bioinformatic analysis was performed using the OmicStudio tools at https://www.omicstudio.cn/tool (accessed on 21 March 2022)).

### 4.9. Quantitative Real-Time PCR

To validate the transcriptome sequencing results, real-time quantitative polymerase chain reaction (qRT-PCR) was used to determine the expression levels of three key genes, namely, *psaD*, *sodA*, and *por*. The qRT-PCR methods are detailed in the Supplementary Information.

### 4.10. Statistical Analysis

The experimental data were analyzed using one-way ANOVA (*p* < 0.05) and least significant differences between control and treatment groups were determined by Duncan’s multiple-range test (*p* < 0.05) in IBM SPSS 23.0. Standard errors were calculated using Excel software. Data were the results of multiple parallel experiments.

## 5. Conclusions

Exposure to 17α-EE_2_ significantly inhibited the growth of *C. pyrenoidosa*. It reduced the cell density, damaged the cell structure, decreased the content of photosynthetic pigment, and diminished the photosynthetic system activity. It down-regulated the relevant photosynthetic genes and ultimately impaired photosynthesis. At the same time, 17a-EE_2_ induced oxidative stress in algal cells, causing oxidative damage. This was followed by an increase in ROS and MDA levels and antioxidant enzyme activity in *C. pyrenoidosa*, stimulating the antioxidant system and up-regulating some of the relevant regulatory genes as a means of providing protection against oxidative damage. Transcriptomic analysis showed that the common pathways affected in algal cells exposed to different treatment concentrations of 17a-EE_2_ were photosynthesis and DNA synthesis and repair. However, certain specific pathways exhibited different behaviors at different concentrations. The pathways of ribosome biogenesis in eukaryotes, photosynthesis, and oxidative phosphorylation were affected at low doses of 17a-EE_2_, while that of ribosome biogenesis in eukaryotes, porphyrin and chlorophyll metabolism, nitrogen metabolism, and DNA damage repair were affected at moderate doses and higher doses of 17a-EE_2_. This study contributes to the understanding of the molecular mechanisms underlying the response of *C. pyrenoidosa* to 17α-EE_2_ and provides a dataset for a relevant ecological risk assessment.

## Data Availability

Not applicable.

## Supplementary Materials

The following supporting information can be downloaded at: https://www.mdpi.com/article/10.3390/ijms23073583/s1.
