# Supplementary material for: Transcriptomic and Physiological Responses of Chlorella pyrenoidosa during Exposure to 17α-Ethinylestradiol
Source: Int J Mol Sci. 2022 Mar 25;23(7):3583. doi: 10.3390/ijms23073583 (PMC8999151; doi:10.3390/ijms23073583)
Supplement: Supplementary file 1 [file ijms-23-03583-s001.zip › ijms-1572632-supplementary.pdf]

International Journal of *Molecular Sciences*

MDPI

*Supplementary Material*

# Transcriptomic and Physiological Responses of *Chlorella pyrenoidosa* during Exposure to 17α-Ethinylestradiol

**Annexed Table**

| **Table Captions:** | |
|---|---|
| **Table S1.** | Formulae and terms used in the analysis of the O-J-I-P fluorescence-induction dynamics curve. |
| **Table S2.** | Variance of ABS/RC, RC/$CS_0$, $ET_0$/ABS, $\psi_O$, RC/$CS_0$, $DI_0$/RC, $ET_0$/RC, and $TR_0$/RC in response to exposure time under at different 17α-$EE_2$ concentrations. |
| **Table S3.** | The lists of primers used in real-time-quantitative PCR. |
| **Table S4.** | The Lists of DEGs belonging to different pathways in the LD, MD, and HD groups. |
| **Figure captions:** | |
| **Fig. S1** | The transcript abundance of genes related to *psaD*, *sodA*, and *por* in cultures of *C. pyrenoidosa* exposure to 17α-$EE_2$. |
| **Fig. S2** | Scatter plots of KEGG enrichment pathway in the LD, MD, and HD groups. |
| **Fig. S3** | KEGG-annotated changes in gene expression for photosynthesis pathways in the LD group. |
| **Fig. S4** | KEGG-annotated changes in gene expression for oxidative phosphorylation pathways in the LD group. |
| **Fig. S5** | KEGG-annotated changes in gene expression for ribosome biogenesis in eukaryotes pathways in the LD group. |
| **Fig. S6** | KEGG-annotated changes in gene expression for ribosome biogenesis in eukaryotes pathways in the MD group. |
| **Fig. S7** | KEGG-annotated changes in gene expression for pyrimidine metabolism pathways in the MD group. |
| **Fig. S8** | KEGG-annotated changes in gene expression for porphyrin and chlorophyll metabolism pathways in the MD group. |
| **Fig. S9** | KEGG-annotated changes in gene expression for nitrogen metabolism pathways in the MD group. |
| **Fig. S10** | KEGG-annotated changes in gene expression for DNA replication pathways in the MD group. |
| **Fig. S11** | KEGG-annotated changes in gene expression for DNA replication pathways in the HD group. |
| **Fig. S12** | KEGG-annotated changes in gene expression for mismatch repair pathways in the HD group. |
| **Fig. S13** | KEGG-annotated changes in gene expression for homologous recombination pathways in the HD group. |

**Table S1.** Formulae and terms used in the analysis of the O-J-I-P fluorescence-induction dynamics curve.

| Formulae and Terms | Illustrations |
|---|---|
| O-step, K-step, J-step, I-step, P-step | Fluorescence intensity at t=50 μs, 30 μs, 2 ms, 30 ms, and the highest peak of fluorescence, respectively |
| *ABS/RC* | Light energy absorbed per unit reaction center |
| *Fv/Fm* | The maximum quantum yield for primary photochemistry |
| $ET_0/ABS$ | Quantum yield for electron transport (at t=0) |
| $\psi_O$ | Probability that a trapped exciton moves an electron into the electron transport chain beyond $QA^-$ (at t=0) |
| $RC/CS_0$ | Density of RCs (QA-reducing PSII reaction centers) |
| $DI_0/RC$ | Dissipated energy flux per RC (at t=0) |
| $ET_0/RC$ | Electron transport flux per RC (at t=0) |
| $TR_0/RC$ | Trapped energy flux per RC (at t=0) |

**Table S2.** Variance of ABS/RC, $TR_0$/RC, $ET_0$/RC, $DI_0$/RC, Fv/Fm, RC/$CS_0$, $\psi_O$, and $ET_0$/ABS in response to exposure time under different 17α-$EE_2$ concentrations. Values represent mean ± standard error. The letters (a–f) indicate homogeneity of different treatments (P < 0.05).

| Photosynthetic Parameters | Time（h） | CK | LD | MD | HD |
|---|---|---|---|---|---|
| ABS/RC | 24 | 2.52±0.022a | 2.64±0.054c | 2.55±0.022ab | 2.60±0.021bc |
| | 48 | 3.63±0.601a | 3.37±0.036a | 3.36±0.024a | 3.46±0.055a |
| | 72 | 2.99±0.037a | 2.92±0.023a | 2.94±0.041a | 3.00±0.075a |
| | 96 | 3.14±0.008a | 3.07±0.018b | 3.07±0.016b | 3.14±0.040a |
| TRo/RC | 24 | 1.59±0.003a | 1.66±0.043b | 1.64±0.015b | 1.59±0.020a |
| | 48 | 2.33±0.052a | 2.35±0.014a | 2.33±0.024a | 2.37±0.041a |
| | 72 | 2.20±0.029a | 2.16±0.013a | 2.15±0.028a | 2.14±0.036a |
| | 96 | 1.59±0.022a | 1.66±0.011b | 1.64±0.010bb | 1.59±0.033b |
| ETo/RC | 24 | 0.70±0.007a | 0.72±0.016a | 0.69±0.020a | 0.62±0.005b |
| | 48 | 1.14±0.044a | 1.11±0.005a | 1.03±0.055b | 0.96±0.026c |
| | 72 | 0.95±0.009a | 1.02±0.014b | 0.99±0.029ab | 1.01±0.053b |
| | 96 | 0.70±0.009a | 0.72±0.017b | 0.69±0.050b | 0.62±0.067a |
| DIo/RC | 24 | 0.92±0.018a | 0.97±0.018b | 0.91±0.020a | 1.01±0.010c |
| | 48 | 1.30±0.566a | 1.02±0.052a | 1.02±0.040a | 1.10±0.209a |
| | 72 | 0.79±0.027a | 0.77±0.013a | 0.79±0.029a | 0.86±0.039b |
| | 96 | 0.92±0.022a | 0.97±0.013a | 0.91±0.023a | 1.01±0.015b |
| Fv/Fm | 24 | 0.63±0.004a | 0.63±0.006a | 0.64±0.006b | 0.61±0.004c |
| | 48 | 0.64±0.104a | 0.70±0.006a | 0.70±0.005a | 0.68±0.007a |
| | 72 | 0.74±0.007a | 0.74±0.003a | 0.73±0.007a | 0.71±0.006b |
| | 96 | 0.73±0.007a | 0.72±0.003ab | 0.72±0.006b | 0.70±0.004c |
| RC/Cso | 24 | 62.42±3.551a | 58.03±2.610a | 52.09±1.816b | 48.523±2.099 b |
| | 48 | 128.05±9.343a | 119.21±2.355a | 98.54±2.978b | 78.57±1.616c |
| | 72 | 84.67±1.526a | 86.13±2.466a | 73.44±2.723b | 58.65±2.972c |
| | 96 | 102.65±4.231a | 103.40±4.691a | 89.15±5.479b | 70.67±4.966c |
| ψo | 24 | 0.42±0.005a | 0.43±0.004ab | 0.42±0.015b | 0.39±0.007c |
| | 48 | 0.49±0.011a | 0.47±0.004a | 0.44±0.026b | 0.40±0.016c |
| | 72 | 0.46±0.009a | 0.52±0.006b | 0.51±0.017b | 0.47±0.018b |
| | 96 | 0.43±0.008a | 0.47±0.007b | 0.46±0.024b | 0.47±0.034a |
| ETo/ABS | 24 | 0.32±0.004a | 0.35±0.001a | 0.34±0.010a | 0.34±0.003b |
| | 48 | 0.33±0.049a | 0.38±0.004a | 0.37±0.019a | 0.33±0.012a |
| | 72 | 0.33±0.005a | 0.38±0.005b | 0.37±0.010b | 0.33±0.011b |
| | 96 | 0.32±0.003a | 0.35±0.004b | 0.34±0.015b | 0.34±0.022a |

**Table S3.** The lists of primers used in real-time-quantitative PCR.

| Gene Name | Forward Primers (5′-3′) | Reverse Primers (5′-3′) |
|---|---|---|
| *psaD* | GATTTTACGGCAGCAAGA-GAGC | ACAGAACGAGGGCGACCAT |
| *sodA* | AAGGTCTACACCAC-CGAGCG | GTGCCTTTCGGATTTCGTTG |
| *por* | CTTCTTT-GCCCTCTCTGGTGTG | TACGACCTTCTCGCCCTTCTT |
| *18s* | CAGCCTGCTAAA-TAGTCACGG | CAGAACATCTAAGGGCATCACA |

Abbreviation: *psaD*, Photosystem I P700 chlorophyll a apoprotein A2; *sodA*, manganese superoxide dismutase; *por*, pyruvate: ferredoxin (flavodoxin) oxidoreductase.

**Table S4.** The lists of DEGs belonging to different pathways in the LD, MD, and HD groups.

| Pathway | Gene Name | KO Entry | log2FC | *P*-Value | Description |
|---|---|---|---|---|---|
| Photosynthesis | *psbA* | K02703 | -3.35 | 0.00039 | Photosystem II protein D1 |
| | *psbB* | K02704 | -2.99 | 0.00060 | Photosystem II 47 kDa protein |
| | *psbC* | K02705 | -3.06 | 0.00033 | Photosystem II protein |
| | *psbE* | K02707 | -2.57 | 0.00051 | Cytochrome b559 alpha subunit of photosystem II |
| | *psbI* | K02710 | -2.38 | 0.00049 | Photosystem II reaction center protein |
| | *Psb28* | K08903 | 0.55 | 0.00040 | Photosystem II reaction center psb28 chloroplastic |
| | *psaD* | K02692 | 2.03 | 0.00041 | Photosystem I reaction center subunit chloroplastic |
| | *petJ* | K08906 | -0.21 | 0.00042 | Cytochrome c6 |
| | *petB* | K02635 | -3.02 | 0.00117 | Cytochrome b6 (chloroplast) |
| | *petF* | K02639 | -0.25 | 0.00003 | Chloroplast precursor |
| | *crtz* | K15746 | -0.29 | 0.00022 | Beta-carotene hydroxylase |
| Oxidative phosphorylation | *atpA* | K02111 | -3.14 | 0.00020 | ATP synthase CF1 alpha subunit |
| | *atpG* | K02109 | -0.03 | 0.00091 | CF0 ATP synthase subunit II precursor |
| | *coxI* | K02256 | -2.48 | 0.00044 | Cytochrome c oxidase subunit I |
| | *sodA* | K04564 | 2.13 | 0.00889 | Manganese superoxide dismutase |
| | *por* | K02956 | 1.17 | 0.00004 | Pyruvate:ferredoxin (flavodoxin) oxidoreductase |
| Ribosome biogenesis in eukaryotes | *Kin14n* | K14565 | -1.45 | 0.00067 | P-loop containing nucleoside triphosphate hydrolase |
| Nitrogen metabolism | *nita* | K10534 | -2.96 | 0.00001 | Nitrate reductase |
| | *gdh2* | K00261 | -2.11 | 0.00045 | Glutamate dehydrogenase |
| DNA replication | *mcm4* | K02212 | -2.00 | 0.00032 | DNA replication licensing factor |
| | *rpa1D* | K07466 | -1.99 | 0.00011 | Replication A 70 kDa DNA-binding subunit B |
| Mismatch repair | *msh1* | K08737 | 1.11 | 0.00001 | DNA mismatch repair protein MSH1, mitochondrial |
| | *exo1* | K10746 | -2.28 | 0.00003 | Exonuclease 1 |
| | *mus1* | K08735 | -1.56 | 0.00007 | DNA mismatch repair |

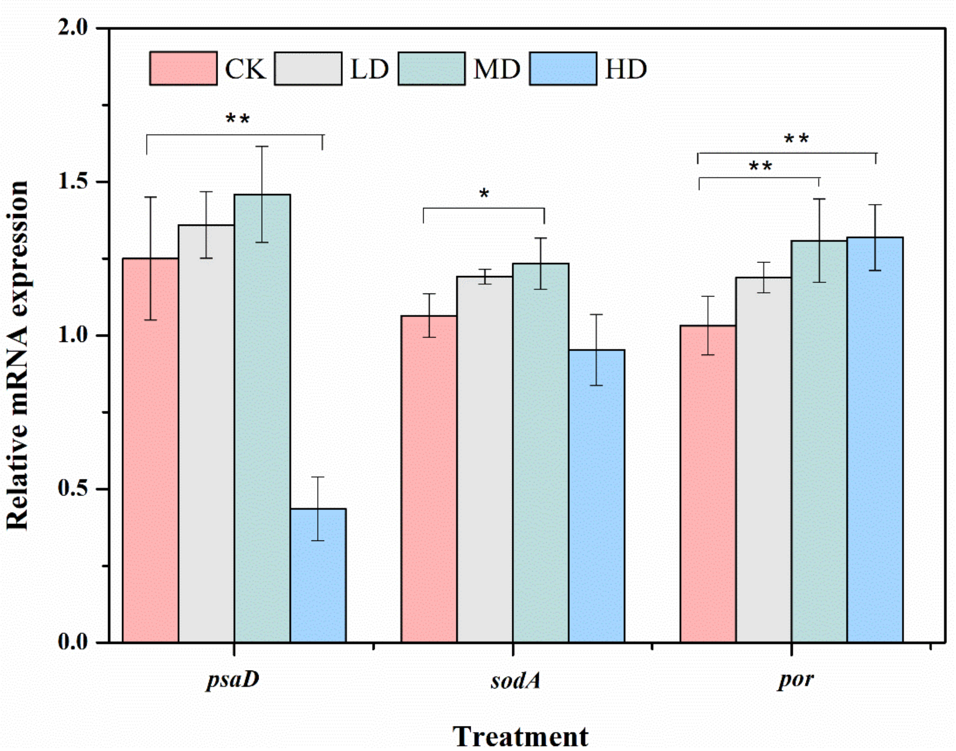


**Figure S1.** The transcript abundance of genes related to *psaD*, *sodA*, and *por* in cultures of *C. pyrenoidosa* exposed to 17$\alpha$-$EE_2$. Error bars indicate mean ± SE. (*) and (**) represent statistically significant differences with respect to the values of control cultures at $p < 0.05$ and at $p < 0.01$ levels, respectively.

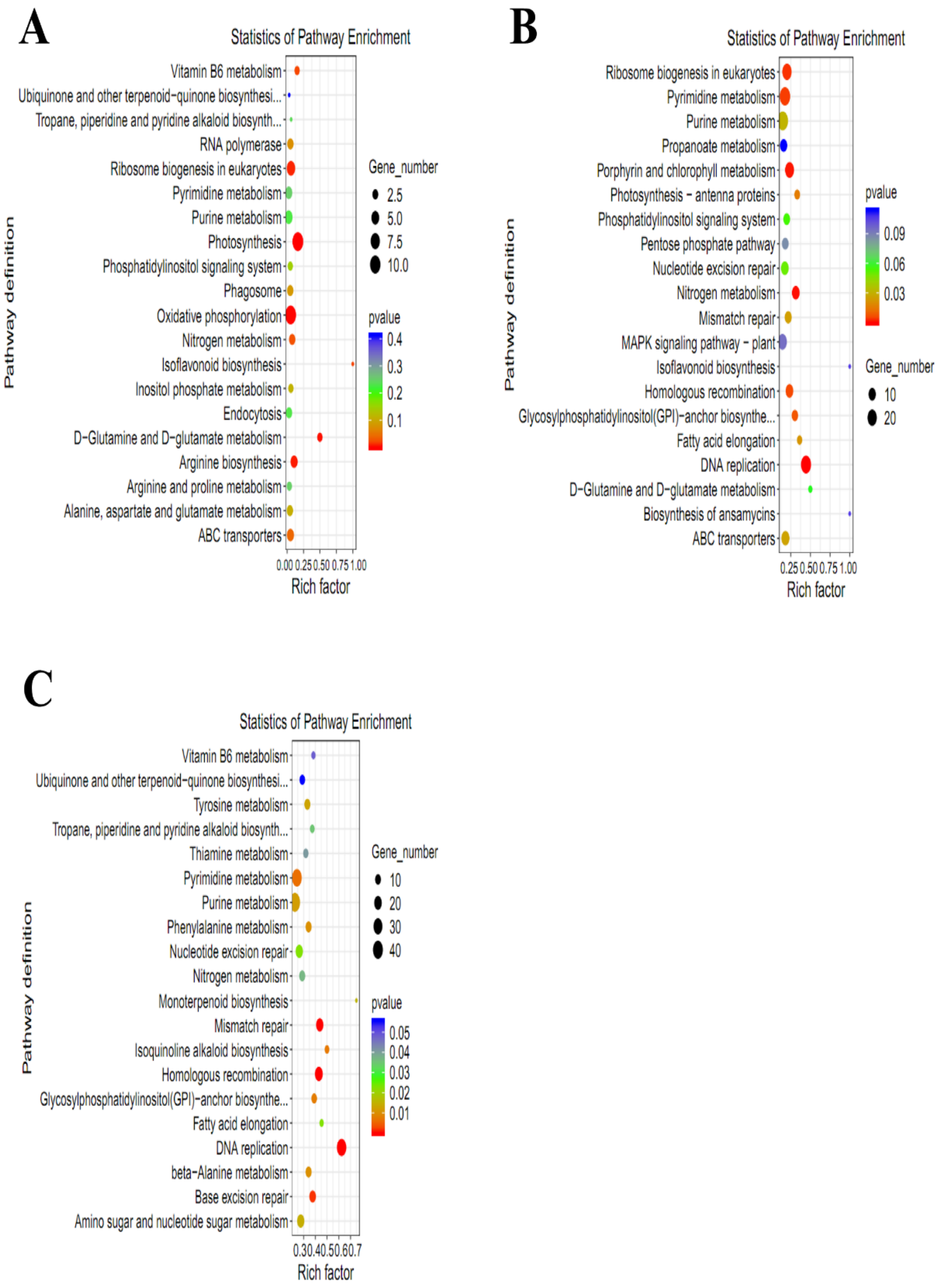


**Figure S2.** Scatter plots of KEGG enrichment pathway in the LD (**A**), MD (**B**), and HD (**C**) groups.

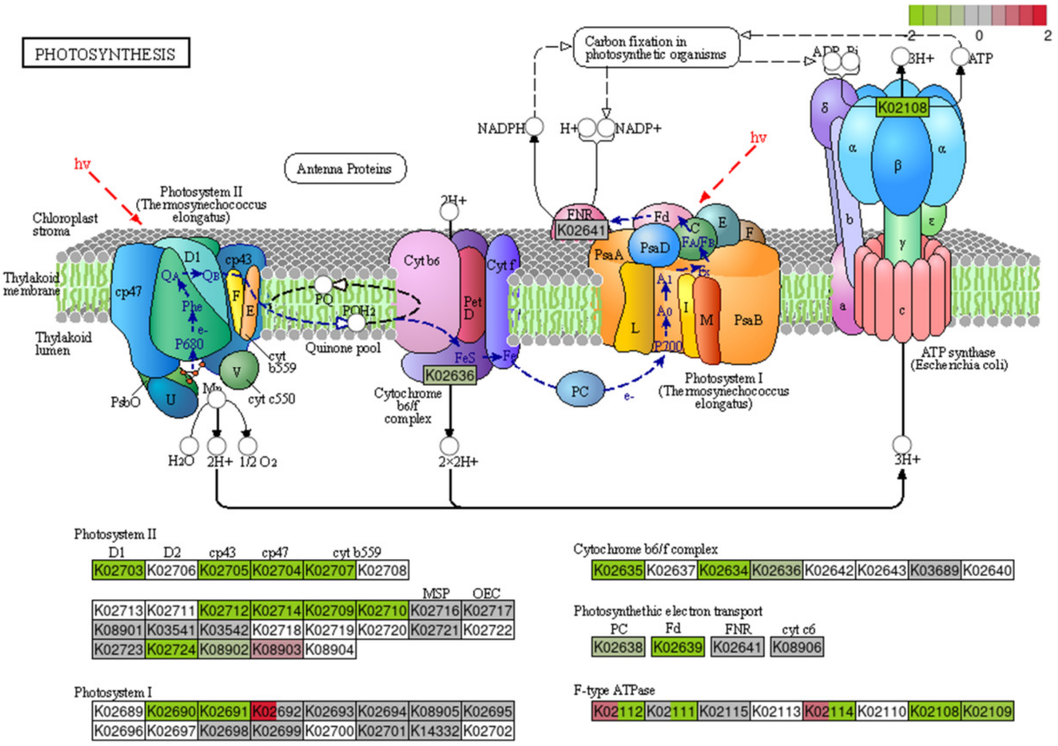


**Figure S3.** KEGG-annotated changes in gene expression for photosynthesis pathways in the LD group (red square: compared with CK, significantly up-regulated in LD; green square: compared with CK, significantly down-regulated in LD).

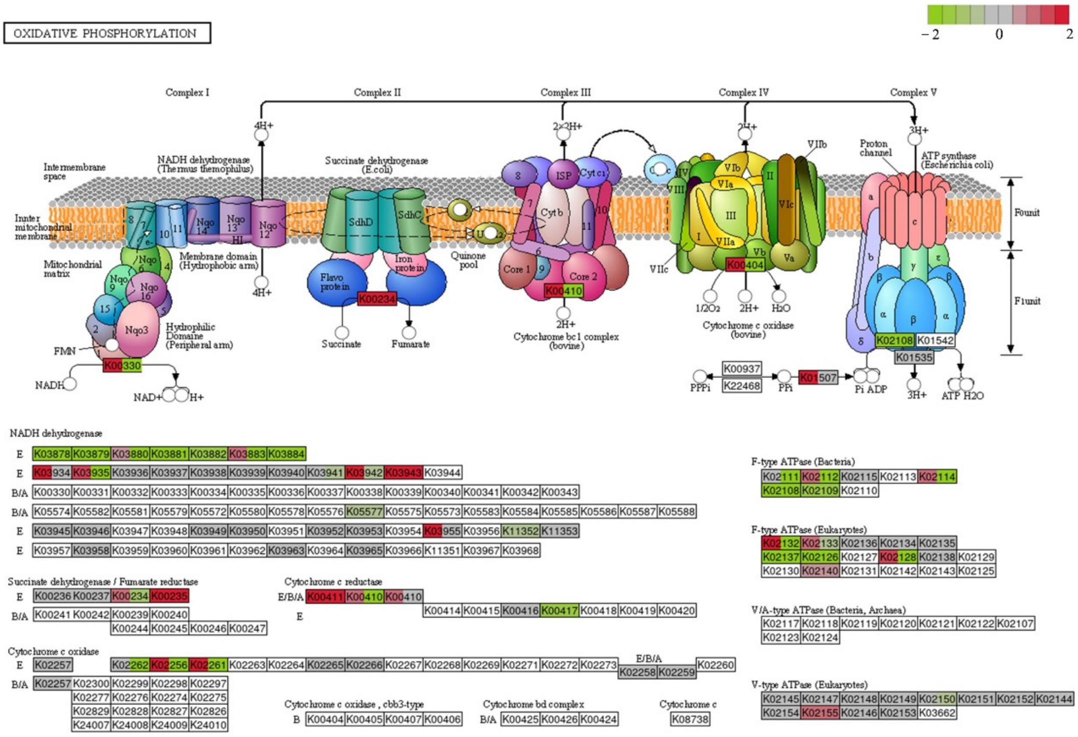


**Figure S4.** KEGG-annotated changes in gene expression for oxidative phosphorylation pathways in the LD group (red square: compared with CK, significantly up-regulated in LD; green square: compared with CK, significantly down-regulated in LD).

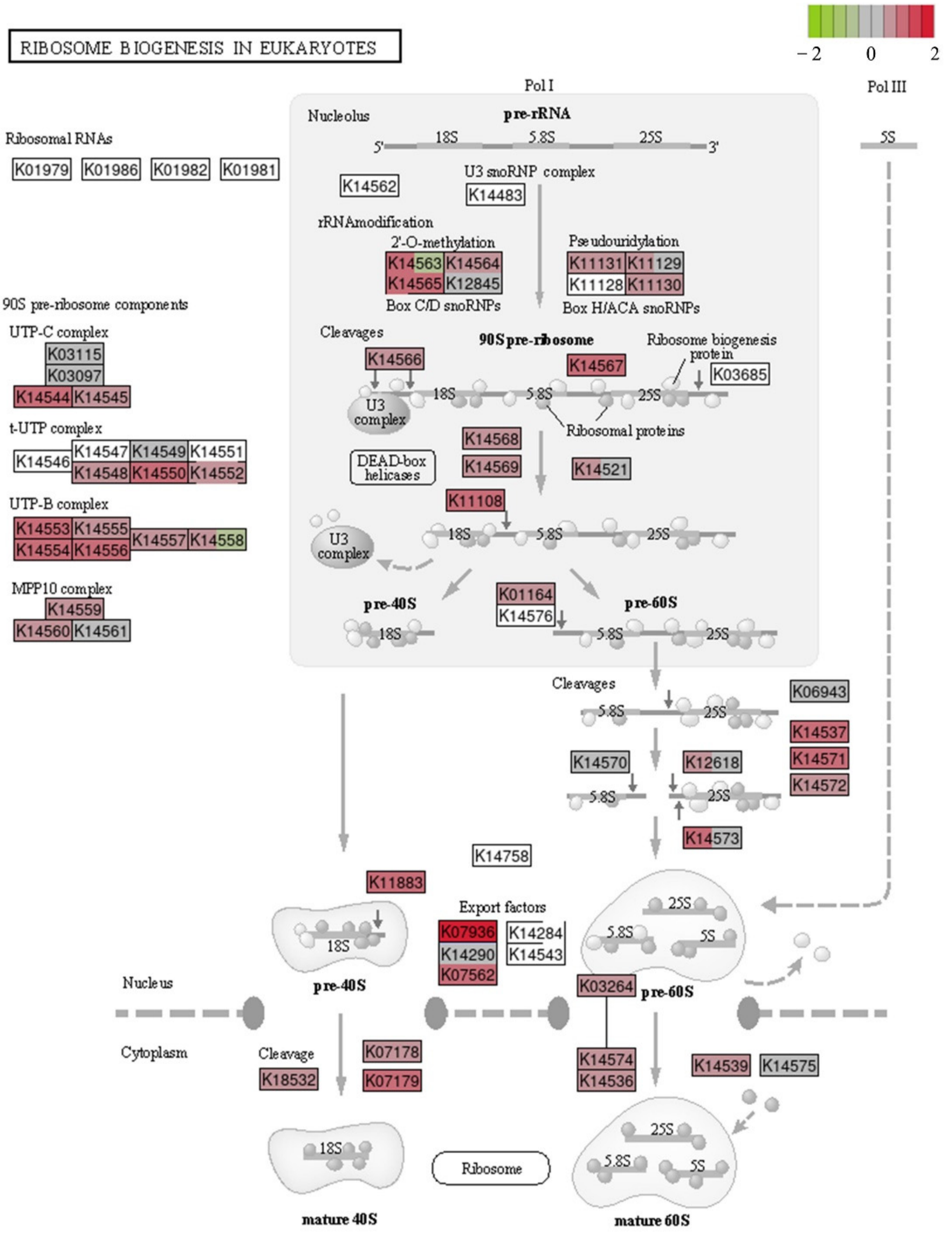


**Figure S5.** KEGG-annotated changes in gene expression for ribosome biogenesis in eukaryotes pathways in the LD group (red square: compared with CK, significantly up-regulated in LD; green square: compared with CK, significantly down-regulated in LD).

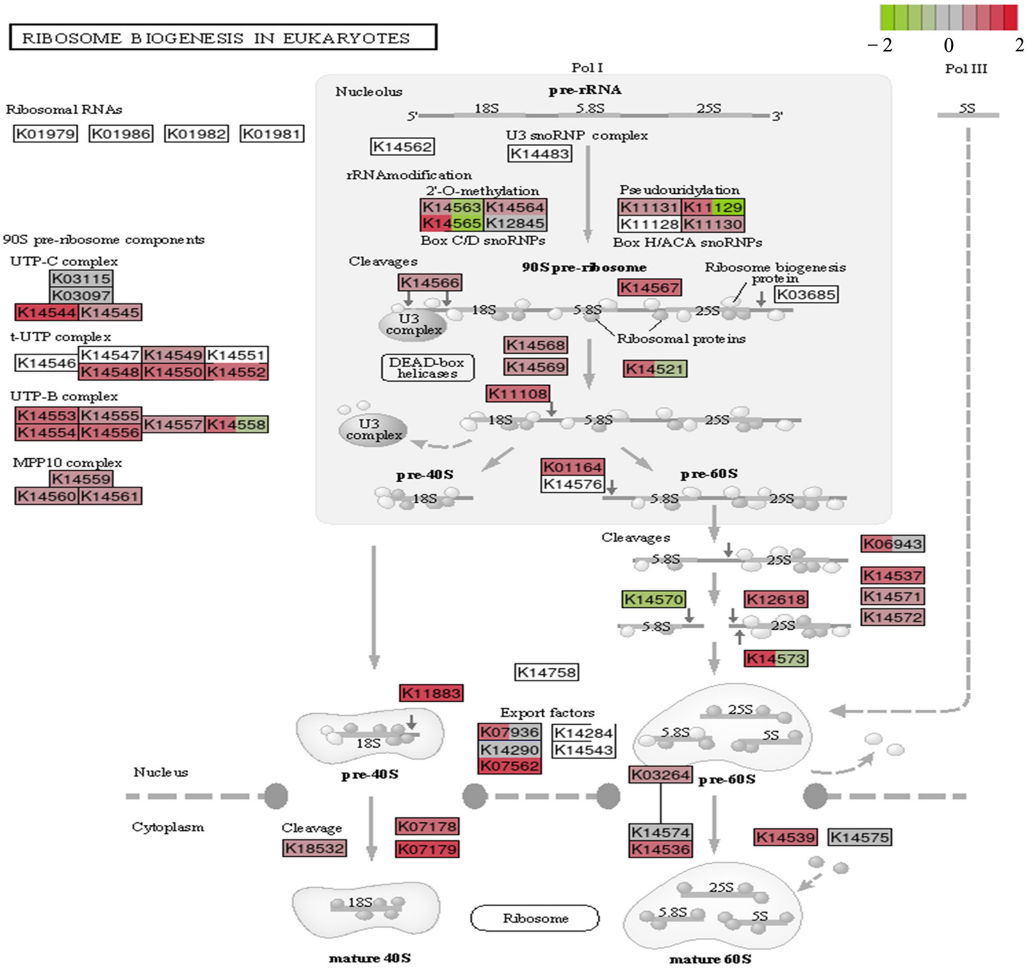


**Figure S6.** KEGG-annotated changes in gene expression for ribosome biogenesis in eukaryotes pathways in the MD group (red square: compared with CK, significantly up-regulated in MD; green square: compared with CK, significantly down-regulated in MD).

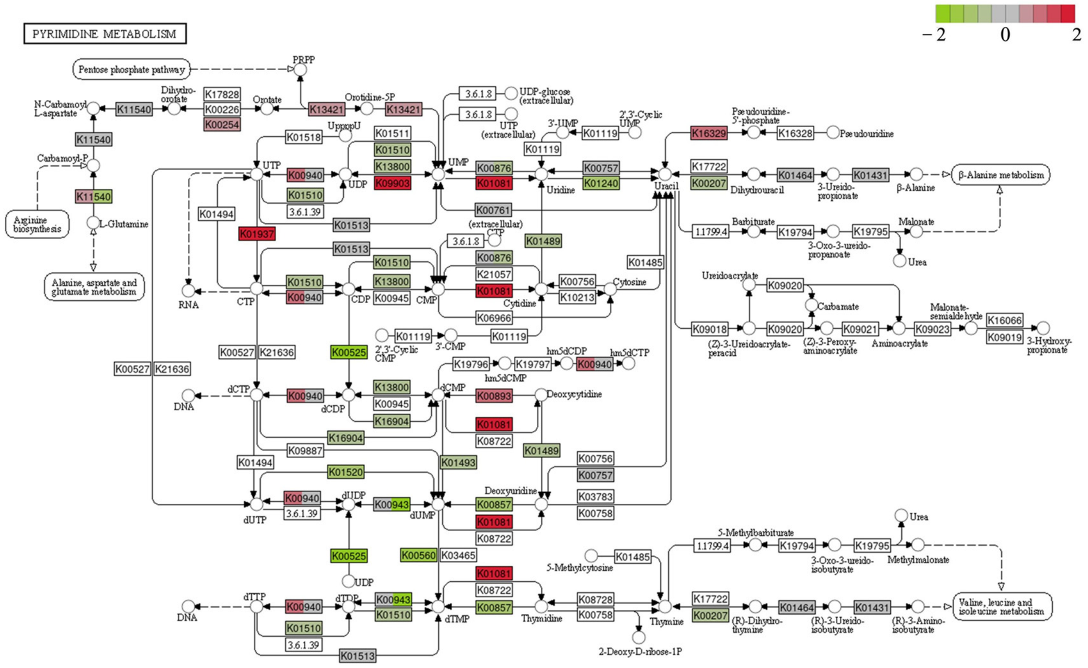

**Figure S7.** KEGG-annotated changes in gene expression for pyrimidine metabolism pathways in the MD group (red square: compared with CK, significantly up-regulated in MD; green square: compared with CK, significantly down-regulated in MD).

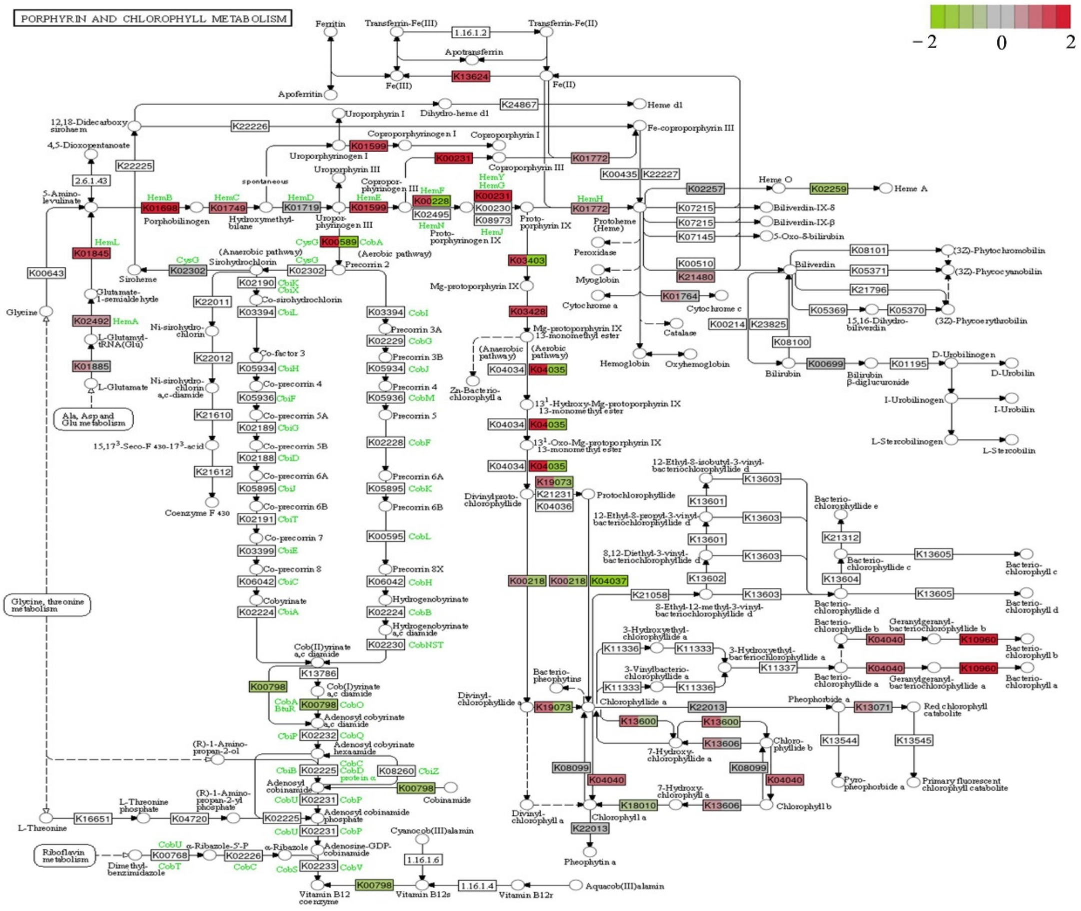


**Figure S8.** KEGG-annotated changes in gene expression for porphyrin and chlorophyll metabolism pathways in the MD group (red square: compared with CK, significantly up-regulated in MD; green square: compared with CK, significantly down-regulated in MD).

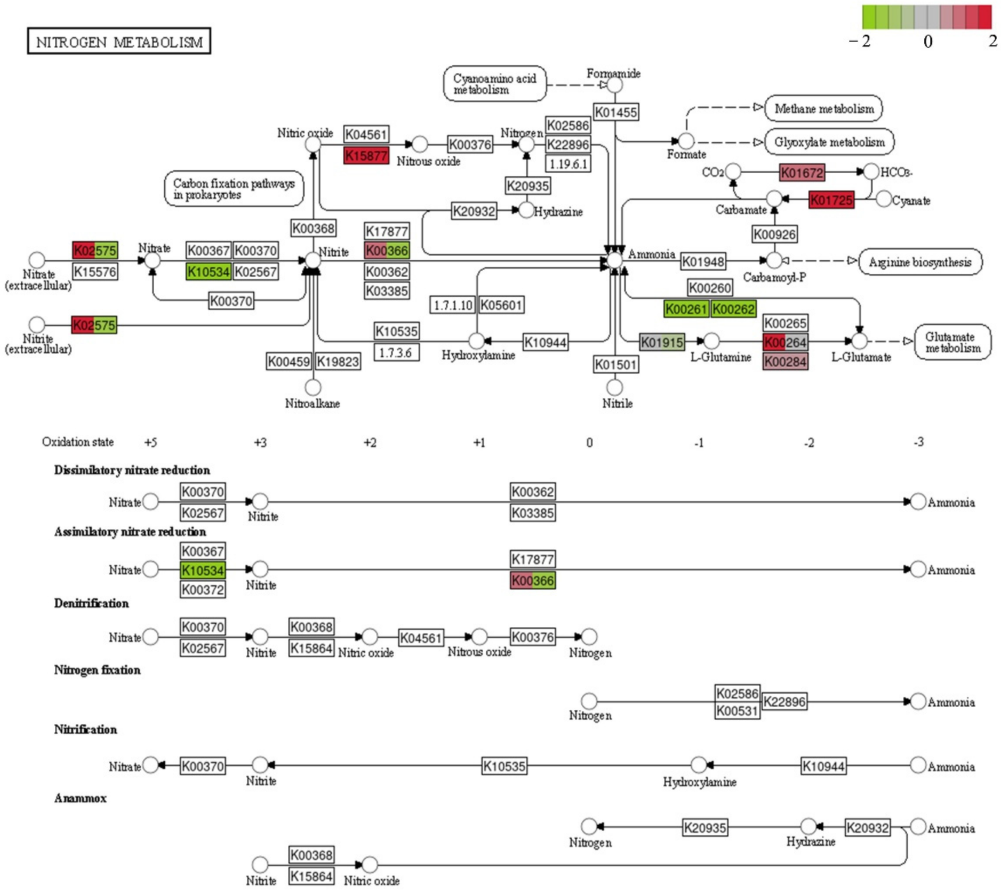


**Figure S9.** KEGG-annotated changes in gene expression for nitrogen metabolism pathways in the MD group (red square: compared with CK, significantly up-regulated in MD; green square: compared with CK, significantly down-regulated in MD).

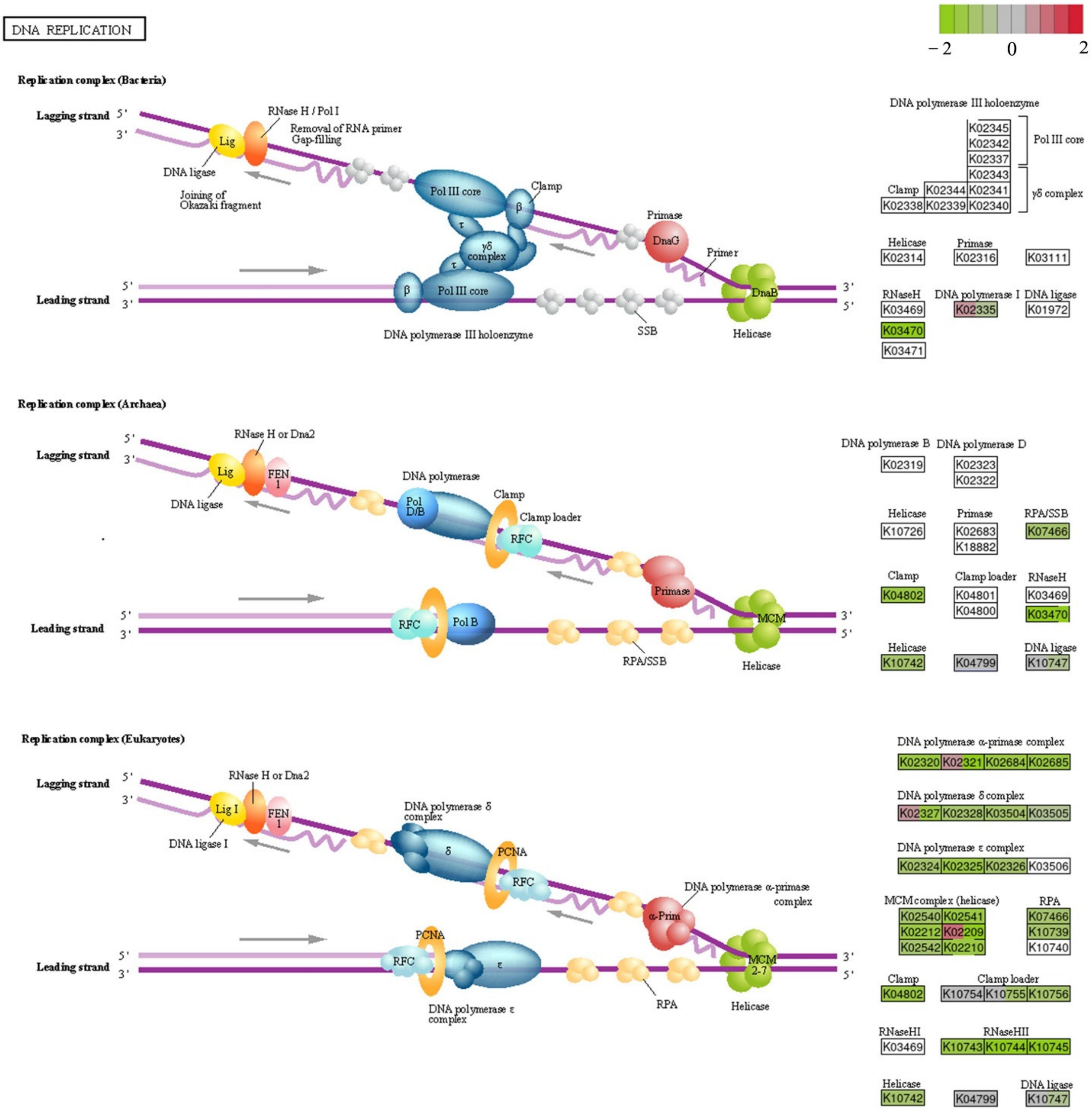


**Figure S10.** KEGG-annotated changes in gene expression for DNA replication pathways in the MD group (red square: compared with CK, significantly up-regulated in MD; green square: compared with CK, significantly down-regulated in MD).

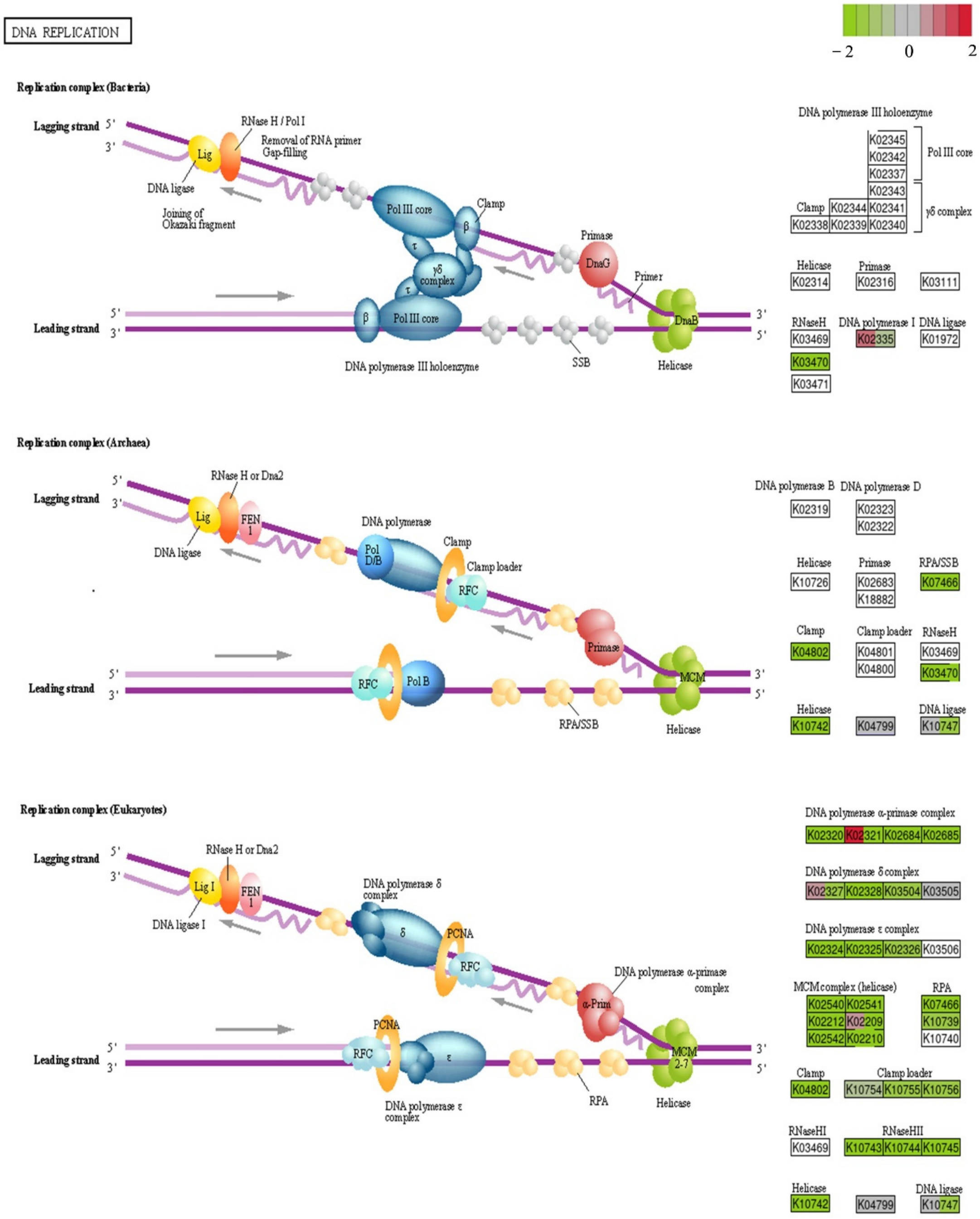


**Figure S11.** KEGG-annotated changes in gene expression for DNA replication pathways in the HD group (red square: compared with CK, significantly up-regulated in HD; green square: compared with CK, significantly down-regulated in HD).

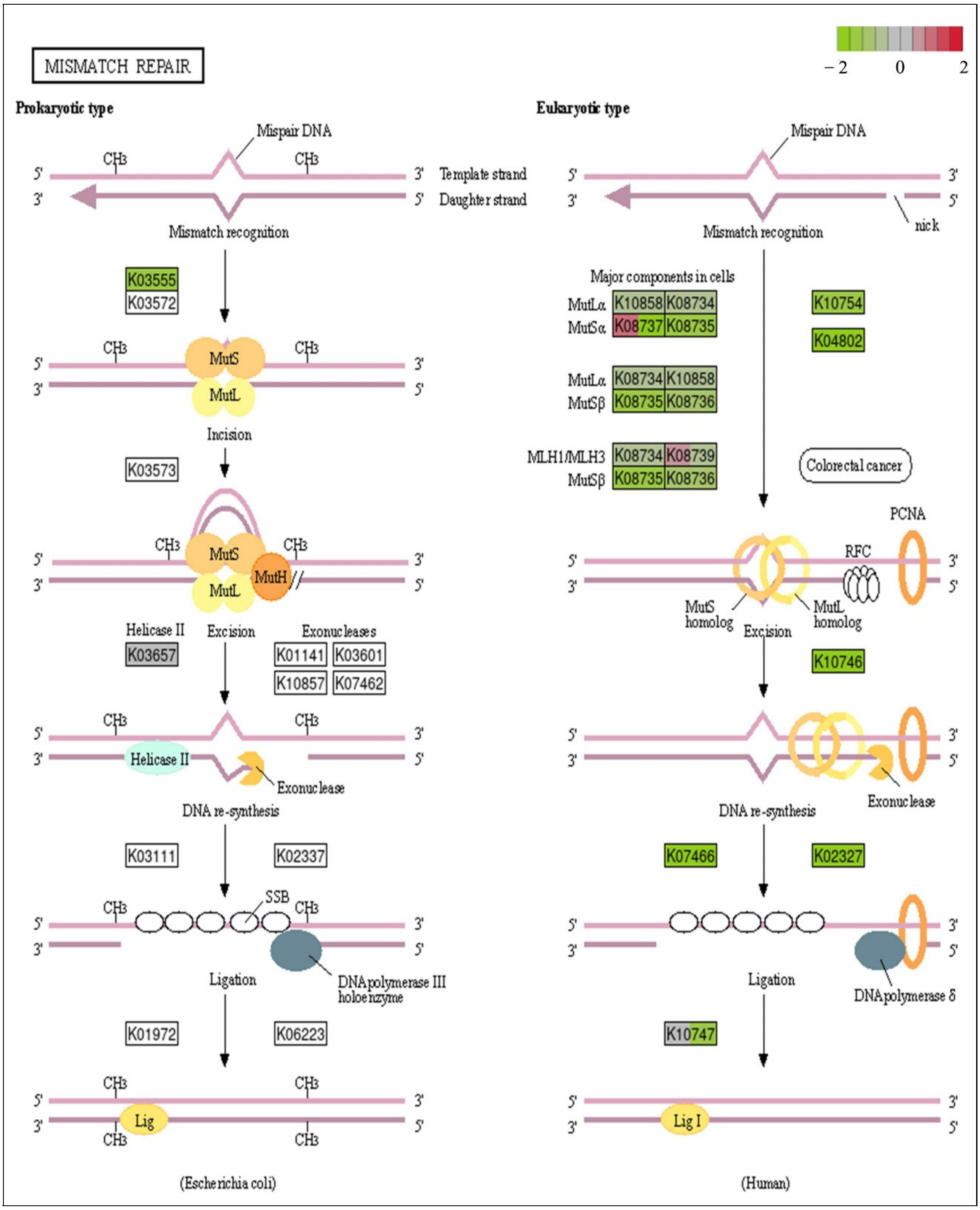


**Figure S12.** KEGG-annotated changes in gene expression for mismatch repair pathways in the HD group (red square: compared with CK, significantly up-regulated in HD; green square: compared with CK, significantly down-regulated in HD).

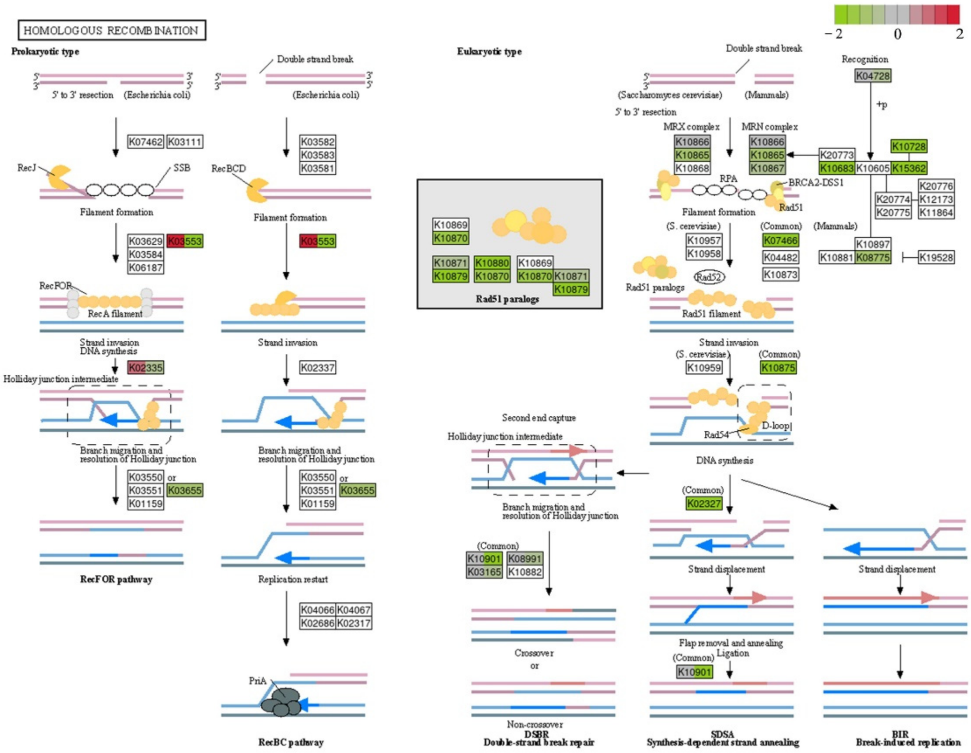

**Figure S13.** KEGG-annotated changes in gene expression for homologous recombination pathways in the HD group (red square: compared with CK, significantly up-regulated in HD; green square: compared with CK, significantly down-regulated in HD).
